# Off-farm employment increases women's empowerment: Evidence from rice farms in the Philippines

**DOI:** 10.1016/j.jrurstud.2019.09.002

**Published:** 2019-10

**Authors:** Rio Maligalig, Matty Demont, Wendy J. Umberger, Alexandra Peralta

**Affiliations:** a Centre for Global Food and Resources, Faculty of the Professions, The University of Adelaide, Level 6 NEXUS 10 Tower, 10 Pulteney Street, SA 5005, Australia; b Agri-food Policy Platform, International Rice Research Institute (IRRI), DAPO Box 7777, Metro Manila 1301, Philippines

**Keywords:** Intrahousehold decision making, Field experiment, SDGs, Women empowerment, Rice

## Abstract

We examined intrahousehold decision making with respect to household investment in portfolios of future rice varietal trait improvements (VTIs) to increase farm households' livelihoods in Nueva Ecija, Philippines. Investment decisions were elicited using an experimental methodology based on investment games. In the investment game, couples from rice farming households were given the opportunity to invest in public rice breeding. They selected, first individually, and then jointly, a replacement rice variety to improve upon and were then asked to allocate a research endowment fund to a portfolio of VTIs. We developed a novel indicator of women's intrahousehold decision-making power (WIDMP) based on the relative Euclidean distances between the individual and joint VTI portfolios. We found that WIDMP is normally distributed; and that, on average, women had almost equal (48%) decision-making power as men (52%), revealing almost perfect gender equity in investment decision making in rice breeding. Women were slightly more empowered if they were engaged in off-farm employment and were less experienced in farming. More empowered women had a higher discount factor and based their investment decisions on anticipated future trends, rather than current or past experience. The findings not only highlight the importance of considering gender roles in technology design, adoption and extension programs, but also have broader implications in terms of women empowerment programs. Consistent with the Sustainable Development Goals (SDGs), our evidence suggests that education and training programs need to be paired with investments generating off-farm employment opportunities to effectively increase women's bargaining power in the household.

## Highlights

• We develop a novel indicator of women's intrahousehold decision-making power.
• Filipino rice farms exhibit high gender equity in investment decision making in rice breeding.
• Off-farm employment empowers women in investment decision making in rice breeding.
• More empowered women base their investment decisions on anticipated future trends.

## 1 Introduction

Women make significant contributions to the agriculture sector in many developing countries (Food and Agriculture Organization, 2011). Until recently, the common belief was that women in rural areas of developing countries are disadvantaged as they do not have equal access to resources and opportunities compared to men. However, this may not be the case in four Southeast Asian countries: Myanmar, Thailand, Indonesia, and the Philippines. For example, a recent study by Akter et al. (2017) revealed that compared to men, women in these countries actually have the same level of access to resources, including land and production inputs. Further, the authors found that women, may, in fact, have greater control over household income. As a result, they may have relatively higher participation and more influence in household decisions compared to women in other developing countries (e.g., Achandi et al., 2018; Ragsdale et al., 2018; Sell and Minot, 2018).

Farming households, like other households, are faced with multiple decisions. One particular area of decision making in farm production relates to which agricultural technologies to adopt. A key area that has been examined in the adoption literature is farmer preferences for technology attributes. Numerous empirical studies focus on this as adoption decisions are influenced not only by socio-economic, demographic or institutional factors, but also by how farmers perceive the specific traits of the technology (Adesina and Baidu-Forson, 1995; Adesina and Zinnah, 1993; Pingali et al., 2001; Sall et al., 2000).

Most of the studies that have been conducted on farmer preferences for variety traits have elicited preferences of the household head, either male or female, implicitly assuming that his or her preferences represent that of the whole household (Asrat et al., 2010; Ghimire et al., 2015; Hintze et al., 2003; Horna et al., 2007; Joshi and Bauer, 2006; Pant et al., 2011).

In some previous studies, preferences for variety traits were elicited from both male and female farmers (Dalton et al., 2011; Ortega and Ward, 2016). For example, in previous studies on Participatory Varietal Selection (PVS) programs, female farmers were specifically invited to test and evaluate selected varieties on their own fields (Courtois et al., 2001; Paris et al., 2001, 2008). The aim was to better understand women's preferences in order to develop interventions that could help them make more informed decisions.

Despite common assumptions, empirical evidence from previous studies suggests that farm production decisions, including adoption, are decided within a household with participation of both the husband and the wife (Alwang et al., 2017; Gilligan et al., 2014; Lambrecht et al., 2016; Sumner et al., 2017; Tiruneh et al., 2001; Twyman et al., 2015). However, the adoption literature rarely examines the intrahousehold dynamics and the process of decision making in technology design and adoption.

### 1.1 Study objective

The main objective of this paper is to answer the following question: What are the factors that empower women in intrahousehold decision making on investment in public rice breeding, more specifically on the choice of the rice varietal trait improvements (VTIs) that are needed to improve the farm household's livelihood? To answer this question, we conducted a framed field experiment using the Investment Game Application (IGA) with selected rice farming households in Nueva Ecija, Philippines (Demont et al., 2015).

### 1.2 Contribution to the literature and overview of methods

Our study adds to the literature in three ways. First, we contribute to the growing body of literature on gender and intrahousehold decision making by considering specifically farm households and their intrahousehold preferences for technology attributes. We examine farmer preferences for *both* individual and joint household decision making in the context of improvements in rice variety traits. Second, we contribute methodologically, as we employ a new and innovative experimental investment game to elicit both individual and joint preferences. The information elicited via the investment game provides insights into the dynamics of decision making in farm households. Lastly, we contribute to the adoption literature as we examine the relative influence of the female spouse in joint decisions, a topic which has previously not been given much consideration in past adoption studies.

The Philippines presents an interesting setting to examine the dynamics of intrahousehold decision making. Based on the statistics on the marital status of the population of 15 years old and over, 55% are married with most of the households having a male as head (Philippine Statistics Authority, 2016a). In rural households, resources are typically pooled (Eder, 2006) and husbands usually entrust part or all their income to their wives (Pajaron, 2016; Upadhyay and Hindin, 2007). Generally, wives share the control over these resources with the husband and they decide jointly on how to allocate them. They also decide jointly on household plans and activities. Moreover, in the Philippines, previous research has shown that wives usually have more control in decisions related to household budgeting and/or expenditures (David, 1994; Eder, 2006; Hindin and Adair, 2002). Further, in rice farming households, decisions with respect to household and farm activities are typically jointly made by husbands and wives, although the level of influence a female spouse has in the process still depends on several factors such as education level and on-farm employment (Hwang et al., 2011; Quisumbing, 1994). Within this setting we examine household preferences in the context of joint decision making.

Due to difficulties in observing the actual farm household decision-making processes, experimental games can be used to elicit intrahousehold decision making on issues that are relevant to the farm's performance and the household's livelihood. Through experimental methods, one can gain a deeper understanding of the dynamics of intrahousehold decision making and resource allocation (Doss, 2013). For example, Beharry-Borg et al. (2009) used a choice experiment to elicit couples' individual and joint preferences for beach sites to visit in Tobago while on vacation. Dosman and Adamowicz (2006) combined stated and revealed preference techniques to obtain both individual and household choice of vacation sites to visit. Several studies used experimental games with real pay-offs to examine couples' individual and joint decisions. These studies considered how decisions made individually by couples differ from the decisions they made jointly (Bateman and Munro, 2005; Munro et al., 2008) and which spouse has more influence on the joint decision (Carlsson et al., 2012, 2013; de Palma et al., 2011).

Similar to these studies, we employ experimental games with real pay-offs to elicit intrahousehold preferences for rice VTIs. Stated preference methods on future products which are not yet available rely on hypothetical scenarios, which make them prone to hypothetical bias (Hensher, 2010; List and Gallet, 2001; Little and Berrens, 2004; Murphy et al., 2005). Therefore, instead of creating markets for future products and then eliciting preferences in those markets, we move upstream and create an investment market for product development, public rice breeding in this case. Using an experimental methodology based on investment games, we elicit preferences of husbands and wives, individually and jointly, for VTIs that can be generated by public rice breeding programs. Similar to a common investment game where there are two players – a sender and a receiver, the investment game in this study also involves a sender, which is the farmer and a receiver, a public research institution (i.e., the International Rice Research Institute (IRRI) and its national partners).

Farmers were given an endowment fund and were asked to decide how much they would send to the receiver. This amount can essentially be considered to be a proxy for the amount the farmer was willing to contribute to or invest in public breeding research for specific VTIs and can also be considered as an indication of a trusting behaviour (Berg et al., 1995; Kocher et al., 2015). Participant farmers were told that IRRI would return an amount to the farmer depending on the expected performance (risk and return) of the portfolio of VTIs chosen. This can be seen as an indication that the receiver is keeping the trust or reciprocating the sender's trusting behaviour (Berg et al., 1995). By rewarding farmers with a return on their investment, the experiment is less hypothetical than other elicitation methods (such as contingent valuation and discrete choice experiments). Additionally, farmers faced real incentives to contribute to breeding research instead of keeping their entire endowment fund.

## 2 Conceptual approach

### 2.1 Models of household decision making

Early models of household decision making assumed a unitary framework, wherein a household is considered as a single production or consumption unit (Becker, 1965). In this framework, it is assumed that the household pools its resources, such as income, labour, land, and information. The allocation of such resources among household members is assumed to be determined by a single member, who is either motivated by self-interest or altruistic behaviour, and with the assumption that all members share the same preferences (Alderman et al., 1995; Haddad et al., 1997).

However, these assumptions can be misleading since preferences may differ in a household, and resources are not necessarily pooled (Attanasio and Lechene, 2002; Duflo and Udry, 2004; Hoddinott and Haddad, 1995; Lundberg et al., 1997). The realisation that the aforementioned assumptions regarding household decision making may be flawed, led to the development of different collective models.

The collective models can be broadly classified into cooperative and non-cooperative models (Alderman et al., 1995; Haddad et al., 1997). There are two types of cooperative models. The first one assumes that household decisions will always lead to pareto-efficient outcomes such that “no one can be made better off without someone being made worse off" (Alderman et al., 1995, p. 5). The second type of cooperative models relies on a game theory model in which a specific bargaining process is used to reach household allocation decisions (Manser and Brown, 1980; McElroy, 1990; McElroy and Horney, 1981).

The second group of collective models, the non-cooperative approach, does not assume that households necessarily attain efficient allocation of resources. Rather, they assume that household members are not obligated to have a binding contract with each other (Alderman et al., 1995).

Ultimately, decision making and allocation of resources within a household are determined by the relative influence or bargaining power of each household member (Quisumbing, 2003). Bargaining power cannot be observed directly; at best it can be represented by different proxies or indicators (Doss, 2013).

Several studies considered the relative contribution to household income, participation in the labour market, and property or asset ownership as key determinants of authority in household decisions (Antman, 2014; Attanasio and Lechene, 2002; Doss, 2006; Doss et al., 2014; Quisumbing and Maluccio, 2003; Swaminathan et al., 2012; Twyman et al., 2015). Other factors such as education (Alwang et al., 2017; Bertocchi et al., 2014), social and political assets (Carlsson et al., 2012, 2013; Orr et al., 2016), and gender institutions and ideology (Bradshaw, 2013; Mabsout and van Staveren, 2010) have also been previously used as proxies. Participation or involvement in the decision-making process is also one of the factors that has been examined in the influence of the bargaining power of an individual within a household (Lépine and Strobl, 2013; Reggio, 2011; Smith et al., 2003).

### 2.2 Conceptual basis

Our conceptual framework is influenced by the insights provided by different models of intrahousehold resource allocation and decision making. We specifically examine the influence of bargaining power indicators on the outcomes of household decision making on investment in improvements in rice variety traits. We examine this through an experimental methodology, which can be used to understand intrahousehold dynamics.

In the study setting, a husband and wife from a rice farming household faced both an individual, and a joint, two-stage decision-making process concerning improvements in rice variety traits. In the first stage, they had to select a replacement rice variety they wanted public rice breeding programs to improve upon. In the second stage, they had to decide which traits of this variety they wanted rice breeding programs to focus on and the levels of VTIs for each trait they wanted these programs to achieve. To do this, they had to allocate an endowment fund to the different variety traits they wanted public rice breeding programs to focus on improving.

Both the husband and wife were aware of the amount of the endowment fund available to them during the individual decision-making process. However, both also had to decide independently on how to allocate the fund. Their individual decisions that were made in the previous round (i.e., on the choice of replacement variety and with respect to VTIs), were not known to their respective spouses. During the joint round, the same amount of endowment fund was provided. However, they needed to decide as a couple on how to allocate this to the VTIs they preferred. No structured guidelines on how to go about the joint decision-making process were set or implemented. These procedures of collective induction are commonly adopted in social psychology literature (e.g., Laughlin and Hollingshead, 1995).

We expected husband and wife to have different preferences for varieties and VTIs, which may have been conditioned or influenced by their different roles (experiences) and responsibilities in the household. Considering this, we focus specifically on the household's joint decision-making outcome. We assume that the joint decision outcome will depend on the bargaining power of each spouse. Therefore, we consider a set of variables likely to affect bargaining power and we examine how they relate to women's intrahousehold decision-making power (WIDMP).

## 3 Experimental approach

### 3.1 Ethics approval

The University of Adelaide's Human Research Ethics Committee approved the study protocols and all data collection instruments (Ethics Approval Number H-2016-010). Written informed consent was obtained from all individual participants prior to the actual experiment.

### 3.2 Study site and sample design

**Fig. 1:**
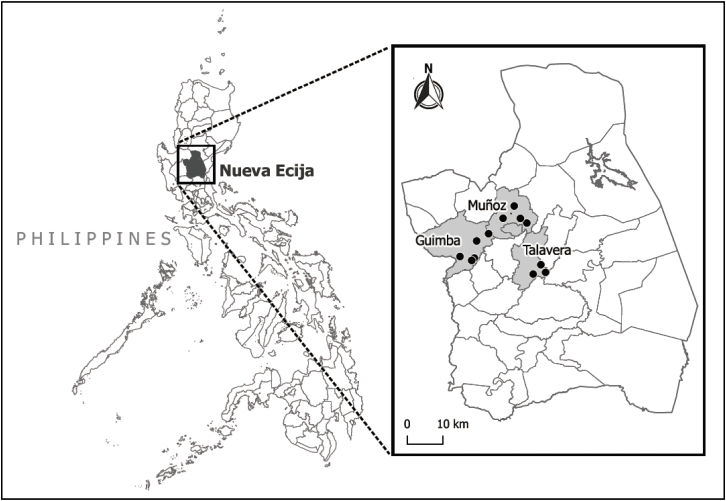
Map of the study site Nueva Ecija with dots indicating the locations of the sampled villages.

The study site is Nueva Ecija, a predominantly irrigated, major rice producing province in the Philippines (Fig. 1). The study site allowed us to capture farmers' preferences for VTIs in both wet and dry seasons. The experiment was conducted in three municipalities - Muñoz, Talavera, and Guimba. A total of 12 villages were then randomly selected. In each village, ten households were randomly chosen from a master list of rice farming households. The master list contained names of farming households in the village that met the following participant selection criteria: (i) both husband and wife should be involved in rice production or rice marketing activities; (ii) the household is planting rice in both wet and dry seasons; and (iii) the household is selling a portion of their rice production.

The randomly selected households were invited to participate in the experiment through the designated local field coordinators in each village. The households were given an invitation letter that explained the details of the research and schedule of the experiment. They were provided with the letter two weeks prior the experiment schedule and were reminded two days before the actual experiment. A total of 122 couples from rice producing households participated in the experiment.

### 3.3 Experimental design and procedures

There were 12 experimental sessions – one for each village selected.^1^ Sessions were conducted in the local language (Filipino) during February 2016. Each session involved the following stages: (i) registration, (ii) introduction of the research team, (iii) explanation of the experiment, (iv) presentation and explanation of the IGA and VTIs, (v) training on the IGA, (vi) six rounds of IGA, (vii) short post-experiment survey, and (viii) payment of returns and closure of the session. Detailed information on the experimental procedures, including the script used by the facilitator, is provided in Appendix A (online Supplementary Material file).

The experimental sessions were framed around a hypothetical context wherein a public rice breeding program received a one million USD investment or grant from a donor. The grant was to be used for the development of an improved rice variety that aimed to replace an existing variety. Farmer participants in the experiments were given the opportunity to become hypothetical shareholders in the public rice breeding program. They were each given a 100 Philippine pesos^2^ (PHP hereafter) endowment fund, which represented a small share of the grant. Participants invested in traits using the graphical interface of a tablet-based IGA (Demont et al., 2015). They were told that they could allocate their share of the grant to several alternative breeding programs for improving varietal traits. The interface of the IGA featured ten VTI bars representing ten different traits that could be improved by the participant. The traits' metric could be improved by using the VTI bars on the IGA interface to increase the corresponding VTI levels from a baseline value up to a target value in steps or increments of 5% (Table 1).^3^ A technical description of IGA is provided in Appendix A.

**Table 1:** Traits and trait-specific metrics used to calibrate the IGA.

Trait	Metric	Baseline	Target
*Grain quality traits*			
Slenderness	Length/width ratio	2.4	3.2
Stickiness	Amylose content (%)	27%	22%
Aroma	Price premium (%) (market benchmark = 100%)	0%	100%
Head rice recovery	% head rice obtained from a sample of paddy	45%	60%
*Stress tolerance traits*			
Lodging tolerance	Crop losses eliminated (%)	20%	80%
Disease resistance	Crop losses eliminated (%)	50%	90%
Insect resistance	Crop losses eliminated (%)	80%	95%
Abiotic stress tolerance	Crop losses eliminated (%)	0%	90%
Reduction in shattering	Crop losses eliminated (%)	80%	95%
*Agronomic trait*			
Earliness	Number of days the duration is shortened	0	14

The experiment included four information treatments that were used to test whether access and exposure to different pieces of forward-looking information affected farmers' investment preferences and intrahousehold decision-making power. The first information treatment was the control, where no information was provided. The second was the market information treatment, which included information on the most preferred rice traits of urban (Metro Manila) consumers and market trends (Custodio et al., 2016).^4^ The third treatment was climate change information. This treatment included information regarding increasing climate variability and the rise in frequency of extreme weather events, which can produce more frequent droughts, floods, and more uncertainty in rainy/wet season onset. The fourth information treatment combined both market information and climate change information.

Prior to administering the IGA, farmers were first trained in the methodology of investing with budget constraints by using the “Training on Investment Game Application” (TIGA). In the TIGA, farmers invested in their optimal dish by adding a vegetable or meat dish to a fixed amount of rice, using a budget amounting to PHP 50. The purpose of the TIGA was to familiarise farmers with the application, particularly in terms of the budget constraint involved and the use of spin buttons on the tablet. It was important that the participants be given the opportunity to use the tablet before the actual game as most of them were not familiar or had not used a tablet before.

The IGA was repeated over six rounds by each participating household. The husband (H) and wife (W) first played the IGA independently and simultaneously for two target seasons, the wet (WS) and the dry (DS). They then played the IGA jointly (J) for two target seasons as well.^5^ During the independent rounds (Round A – H/WS, Round B – W/WS, Round C – H/DS, Round D – W/DS), the husband and the wife were each assigned an agent who facilitated the IGA. In the consensus round (Round E − J/WS, Round F – J/DS), a different agent with a different tablet was assigned per couple. To provide equal opportunity in answering the IGA during the consensus round, husband and wife were given separate stylus pens and the tablet was placed in the middle of their table. However, no further instructions were given and households were free to decide on how to achieve consensus, following similar protocols in experimental economics involving collective induction (Demont et al., 2013).

At the start of each round, farmers were asked to select a ‘replacement variety’ which would provide the base upon which they would invest in public rice breeding research to obtain their ideal variety. The moderator of the experiment explained to participants that the replacement variety does not necessarily need to be their most preferred variety; it could also be a variety they may or may not have previously grown and which could become their ideal variety after some modifications they could propose through their choice of VTIs. After choosing a replacement variety, farmers then selected their preferred VTIs by pulling the VTI investment bars to the level they wanted a trait to be improved.

The experiment included a survey, which was split into two parts. The first part contained general questions on household, farm and marketing characteristics and was administered to the households whenever they were available, i.e., before or after the IGA exercise. The second part was administered after the IGA exercise and included questions on the motivations behind their allocation decisions in IGA and a short quiz (two questions) to verify how well they had understood the experiment.

After all households completed all six rounds (A – F) of the IGA, the final pay-off was determined by the “binding” round, which was randomly selected by rolling a dice, as is commonly done in experimental economics to reduce costs (e.g., Demont et al., 2013; Demont et al., 2012; Lusk and Shogren, 2007; Ward and Singh, 2015). Returns to VTI portfolios were stochastic as they were the result of the joint interaction between the return and the risk function. Returns to investment in public rice breeding research will normally be realized only after a new rice variety is released and adopted. This process will normally take about six to ten years, assuming that accelerated plant breeding methods were used (Collard et al., 2017; Lenaerts et al., 2018, 2019). In our study, breeding investment was framed as an investment with instantaneous return; stochastic returns were calculated in real time and given to the farmer at the end of the experiment. The final pay-off was given in cash and was placed in an envelope before distributing to the couples one at a time. Only the research team had knowledge of the participants' earnings. The participants did not know other participants' earnings. Each household earned on average PHP 1,210, which is roughly equivalent to four daily agricultural wages,^6^ with a maximum of PHP 2,300. On top of the final pay-off, a fixed ‘show-up fee’ amounting to PHP 250 was paid to each household. This was equivalent to around three hours of paid agricultural labour per participant and corresponds to the average time farmers had to give up to participate in the experiment.^7^

## 4 Estimation strategy

We examined the factors that affected wives' influence on intrahousehold decision making on investment. For this, we constructed a measure of the wife's influence on the joint decision with respect to the choice of VTIs, which we refer to as women's intrahousehold decision-making power (WIDMP). Since VTIs are relative to a replacement variety, portfolios of VTIs are only comparable if they refer to the same replacement variety. For this reason, we only included those households whose members happened to have selected the same replacement variety, both individually and jointly. As shown in Table 2, there were five categories depending on whether the joint replacement variety matched with both husband and wife replacement varieties. This was determined at the household level and for the two target seasons. In each season, every household was classified into one of the five categories. Of the 244 observations (122 households × 2 seasons), 175 had the same replacement variety, both individually and jointly (Table 2).

**Table 2:** Agreement in terms of replacement varieties chosen.

Variable	Wet Season		Dry Season		Pooled	
	Frequency	Proportion	Frequency	Proportion	Frequency	Proportion
Joint replacement variety matches both individual replacement varieties	100	82%	75	61%	175	72%
Joint replacement variety matches husband's replacement variety	13	11%	24	20%	37	15%
Joint replacement variety matches wife's replacement variety	3	2%	7	6%	10	4%
Both individual replacement varieties match but joint replacement variety is different	4	3%	9	7%	13	5%
None of the individual and joint replacement varieties match	2	2%	7	6%	9	4%
All	122	50%	122	50%	244	100%

When conducting the IGA exercise, players were essentially constructing portfolios of ten VTIs. These portfolios can be represented as points in a ten-dimensional space. The entire experiment generates six portfolios per household, i.e., four individual (husband and wife over two seasons) and two collective (joint decision over two seasons). We can reasonably assume that, for a given replacement variety, the closer the distance between the individual portfolio constructed by a household member and the collective portfolio agreed upon at household level, the higher the intrahousehold decision-making power of that household member. Therefore, to construct a measure of WIDMP, we first compute the Euclidean distance between the individual and collective portfolio using the following formula:

(1) $$d_{mis}=\sqrt{\sum {\left(VTI_{misk}-VTI_{isk}\right)}^{2}}$$

where *d*_*mis*_ is the Euclidean distance of all the VTIs *k* chosen by spouse *m* of the *i*th household in season *s* to the VTIs *k* chosen jointly in the household. We then construct the indicators for WIDMP and men's intrahousehold decision-making power (MIDMP) as follows:

(2) $$WIDMP_{is}=\frac{d_{his}\;}{d_{wis}\;+\;d_{his}\;},\text{and}\;MIDMP_{is}=\frac{\;d_{wis}}{\;d_{wis}\;+\;d_{his}}=1-WIDMP_{is}$$

These indicators compute the relative distance in VTI portfolio space the other household member has to travel to achieve consensus with the current household member. The more relative distance travelled by the other (current) household member, the more empowered the current (other) household member is in intrahousehold decision making. Hence, WIDMP and MIDMP are relative and exclusive indicators; WIDMP cannot increase unless MIDMP decreases.

WIDMP was subsequently used as the dependent variable in a fractional response model specified as:

(3) $$E\left(WIDMP_{is}|x_{i}\right)=G\left(x_{i}\beta \right)$$

where x are explanatory variables that include individual and household characteristics, and information treatment variables. Following Papke and Wooldridge (1996), G(·) is a nonlinear function satisfying 0≤G(·)≤1 and can be specified as a logistic function, such that the conditional mean of *λ* follows a logit distribution:

(4) $$E\left(WIDMP_{is}│x_{i}\right)=exp\left(x_{i}\beta \right)/\left[1+ex\left(x_{i}\beta \right)\right]$$

Cluster-robust standard errors are calculated at the farm household level. Summary statistics of the dependent and independent variables used in the analysis are presented in Table 3.

**Table 3:** Summary statistics for dependent and independent variables used in the fractional regression model (n = 175).

Variable	Definition	Mean	Std. Dev.	Min	Max
*Dependent variable*					
WIDMP	Women's intrahousehold decision-making power (Equation (2))	0.48	0.2	0	1
*Independent variables*					
Age of wife	Age of wife in years	47.65	10.75	22	73
Age of husband	Age of husband in years	50.67	10.33	21	75
Education of wife	Number of years in school of wife	8.17	2.30	2	14
Education of husband	Number of years in school of husband	8.43	2.66	1	14
Farming experience of wife	Years of rice farming experience of wife	19.35	13.66	0	50
Farming experience of husband	Years of rice farming experience of husband	27.29	12.03	1	55
Time preference of wife^a^	Wife's preference for present values as measured by a discount factor	1.46	2.07	−0.5	9
Time preference of husband	Husband's preference for present values as measured by a discount factor	1.76	5.38	0	49
Risk preference of wife^b^	Willingness of the wife to take risks in investing in farming	4.80	0.42	3	5
Risk preference of husband^b^	Willingness of the husband to take risks in investing in farming	4.94	0.30	2	5
Wife has future perspective	1 – wife bases investment on anticipated future trends, 0 – wife bases investment on past experience	0.55	0.50	0	1
Husband has future perspective	1 – husband bases investment on anticipated future trends, 0 – husband bases investment on past experience	0.38	0.49	0	1
Off-farm employment of wife	1 – wife's primary occupation is in commerce and services, 0 – otherwise	0.30	0.46	0	1
Off-farm employment of husband	1 – husband's primary occupation is in commerce and services, 0 – otherwise	0.06	0.24	0	1
Attendance to training by wife	1 – wife attended agricultural training in the past, 0 – otherwise	0.17	0.38	0	1
Attendance to training by husband	1 – husband attended agricultural training in the past, 0 – otherwise	0.70	0.46	0	1
Membership to organization of wife	1 – wife is member of an organization, 0 – otherwise	0.39	0.49	0	1
Membership to organization of husband	1 – husband is member of an organization, 0 – otherwise	0.45	0.50	0	1
Per capita income	Annual per capita income in ‘000 PHP	17.98	13.82	2	83.33
Percent lease area	Proportion of leased area to total landholdings	0.46	0.49	0	1
Proportion of production sold	Proportion of total production that is sold	0.63	0.23	0	1
Buyer requirement	1 – buyers require certain quality standards, 0 – otherwise	0.63	0.48	0	1
Credit	1 – borrowed cash or other inputs, 0 – otherwise	0.90	0.30	0	1
Market information	1 – exposed to information on market preferences and trends, 0 – otherwise	0.52	0.50	0	1
Climate change information	1 – exposed to information on climate change, 0 – otherwise	0.55	0.50	0	1
Wet season	1 – wet season, 0 – otherwise	0.57	0.50	0	1

a One respondent, who had a negative discount factor (−0.50), preferred to receive a specific amount “today”. When asked how much she preferred to receive after one month for her to prefer to wait (future payout), this respondent answered a lower amount (PHP 500 ) than the amount (PHP 1,000) offered to be given “today”. Thus, her computed discount factor was −0.50 ([500–1000]/1000).

b Risk preference was measured through a Likert scale where 1 – extremely unlikely, 2 – unlikely, 3 – neutral, 4 – likely, 5 – extremely likely.

Previous studies in the literature found that the wife's traits, *relative to her husband's traits,* rather than just her absolute traits, can better capture the strength of her bargaining power (Grossbard-Shechtman and Neuman, 1988). However, since WIDMP = 1 – MIDMP (Equation (2)), both household members' traits can simultaneously affect WIDMP. Therefore, we included both wives' and husbands' socio-demographic characteristics and attitudes. On average, wives were 48 years old. Around 22% of them were older than their husbands. Wives had eight years of formal schooling and about 27% of them were more educated compared to the husbands. Moreover, they had 19 years of rice farming experience and only 7% had more farming experience than the husbands. Only 17% attended agricultural training in the past, while about 39% were members of an organization. We found that only the husband attended agricultural training in more than half of our sample couples, but the majority of them were either both members or both not members of an organization.

We measured time preference through a discount factor, which we estimated from a series of hypothetical questions relating to their preference of receiving a specific amount of cash now or a higher amount in a month's time (Coller and Williams, 1999; Harrison et al., 2002). Wives' discount factor was 146% on average, which was 30% lower than the husband's discount factor. This lower discount factor for wives is supported by the work of Ashraf et al. (2006), which suggests, that due to Filipino women's traditional roles in managing household finances, they are more likely to delay a reward in the distant future and to find solutions to savings problems for the benefit of the household. They are also more likely to take-up commitment savings products compared to men.

When asked about their willingness to take risks in investing in rice farming on a Likert scale, with five representing “extremely likely” and one as “extremely unlikely”, wives' average rating was 4.80. This was 0.14 points lower on average compared to the husbands' rating. This is in line with Croson and Gneezy (2009) who found women are more risk averse compared to men. They suggested that this may be due to differences between men and women in their emotional reaction to risks, confidence in taking risks, and interpretation of risky situations. Furthermore, in the Philippines, due to women's prominent role in managing household finances and income-generating activities (David, 1994; Eder, 2006), women tend to be more risk averse when decisions are likely to affect the welfare of her family.

Moreover, 56% of the wives claimed to take future trends into account in prioritizing traits for improvement. More than one-third of the couples had different considerations in that the wives generally considered the future while the husbands usually considered their past and current farming experience.

The Millennium Development Goals (MDGs) explicitly included “non-agricultural employment” as an indicator for women's empowerment (United Nations, 2008). In the Sustainable Development Goals (SDGs), this issue is further developed and broadened towards social inclusiveness in Goal 2.3: “By 2030, double the agricultural productivity and incomes of small-scale food producers, in particular women, indigenous peoples, family farmers, pastoralists and fishers, including through secure and equal access to land, other productive resources and inputs, knowledge, financial services, markets and opportunities for value addition and non-farm employment”, and in Indicator 8.3.1 “Proportion of informal employment in non-agriculture employment, by sex” (United Nations, 2017, p. 2–8).

Therefore, we included a variable representing whether either spouse was engaged in off-farm employment. Studies show that employment opportunities outside of the farm provide women an outside option or fall-back position that can improve their bargaining power in household decision making (Doss, 2013; Twyman et al., 2015). Off-farm employment allows them to contribute to household income, learn social and other skills, and provide knowledge and information which can help them participate in household decision making. Thus, we hypothesized that wives engaged in off-farm employment (e.g., vegetable or meat/fish vending, managing small shop/store, dressmaking/tailoring) would have higher WIDMP than those not engaged in off-farm employment.

We also included variables related to marketing – production sold and buyer requirement – to account for the wife's important role in post-harvest decisions. Lastly, we included dummy variables on the information treatment to examine whether access and exposure to forward-looking information (market and climate change trends) would affect WIDMP. This treatment acts as a short-term education, training or awareness program that informs farmers about future needs in terms of quality and climate-resilience traits.

## 5 Results

### 5.1 Replacement varieties and VTI portfolios selected

As previously mentioned, we only included in the analysis couples who chose the same replacement variety, both individually and jointly for each season (Table 2). For 72% of the joint observations, the replacement variety selected by husband and wife individually was the same as the replacement variety they selected during the joint round. This suggests that although husbands largely dominate decision making on varietal adoption, wives' varietal preferences tend to match husbands' preferences, perhaps due to their substantial involvement in post-harvest operations (Table 4).

**Table 4:** Participation in crop choice and post-harvest decision making (n = 175).

Variable	Mean	Std. Dev.	Min	Max
*Crop choice*				
What crop to grow in the field	0.01	0.11	0	1
What rice variety to plant	0.02	0.15	0	1
*Post-harvest operations*				
Amount of rice to store or sell	0.10	0.30	0	1
Where to sell rice or other crops	0.06	0.24	0	1
When to sell rice or other crops	0.06	0.23	0	1
Selecting crop types and seed for the next growing season	0.02	0.13	0	1
Who decides how to spend income from crop sale	0.83	0.37	0	1
Where to store seeds	0.02	0.15	0	1

Note: Participation is measured as a binary variable where 1 – wife only or wife dominates in the decision making, 0 – otherwise.

For the remainder of the observations, we found that 19% of the jointly chosen replacement varieties match either the husband's or the wife's replacement variety. Specifically, the joint replacement variety matched the husband's replacement variety in 15% of the observations, and the wife's replacement variety in 4% of the observations. Lastly, we found that in 9% of the cases, couples arrived at a replacement variety outside of the set of individual choices.

Table 5 presents the replacement varieties selected by couples who had total agreement in the choice of replacement variety, both individually and jointly. The table shows that one variety dominated in the wet season, i.e., NSIC Rc222. This inbred variety was identified as the preferred replacement variety by 82% of the couples. As for the dry season, a major variety identified was SL-8H, a hybrid variety. NSIC Rc222 was also one of the dominant replacement varieties identified in the dry season.

**Table 5:** Replacement varieties selected by couples where their joint choice matched both individual choices (n = 175).

Season	Replacement variety	Freq.	Percent
Wet season (n = 100)	NSIC Rc222	86	86
	NSIC Rc216	10	10
	Others	4	4
Dry season (n = 75)	SL-8H	54	72
	NSIC Rc222	17	23
	Others	4	5

**Fig. 2:**
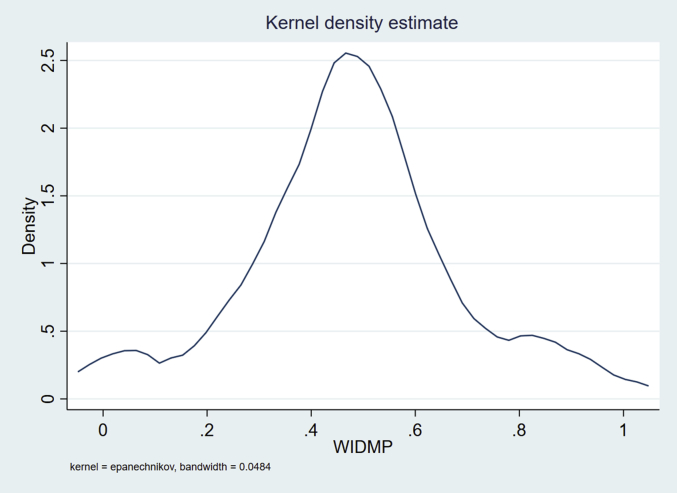
Kernel density of women's intrahousehold decision-making power (WIDMP). A test of normality (Skewness/Kurtosis tests for normality) indicated that WIDMP is normally distributed at the 5% significance level (prob > chi 2 = 0.3748).

Table 6 shows the average portfolios of VTIs constructed by farm households to improve their replacement varieties. The improvements are standardized to a value between 0% and 100% of the distance between the target and baseline VTIs (Table 1). In the wet season, improvements in stress tolerance traits, such as lodging tolerance, disease resistance, and reduction in shattering are prioritized, individually as well as jointly. Improvements in grain quality traits, such as slenderness and head rice recovery receive higher priority in the dry season relative to the wet season. Table 6 also reports some descriptive statistics for intrahousehold decision-making power, which is almost equally shared between husbands (51–53%) and wives (47–49%) in both seasons. Kernel density estimation for WIDMP, our dependent variable of interest, suggests normality (Fig. 2). A Skewness/Kurtosis test for normality confirms that WIDMP is normally distributed at the 5% significance level.

**Table 6:** Average portfolios of VTIs and intrahousehold decision-making power at individual and household level.

VTI	Wet Season (n = 100)						Dry Season (n = 75)					
	Husband		Wife		Joint		Husband		Wife		Joint	
	Mean	Std. Dev.	Mean	Std. Dev.	Mean	Std. Dev.	Mean	Std. Dev.	Mean	Std. Dev.	Mean	Std. Dev.
*Grain quality traits*												
Slenderness	0.11	0.21	0.07	0.15	0.07	0.16	0.12	0.22	0.11	0.25	0.13	0.22
Stickiness	0.06	0.18	0.01	0.05	0.03	0.11	0.01	0.04	0.02	0.12	0.02	0.08
Aroma	0.01	0.05	0.03	0.09	0.01	0.06	0.01	0.04	0.05	0.15	0.04	0.09
Head rice recovery	0.05	0.12	0.05	0.13	0.05	0.12	0.07	0.17	0.10	0.20	0.09	0.18
*Stress tolerance traits*												
Lodging tolerance	0.27	0.28	0.25	0.25	0.25	0.25	0.14	0.23	0.10	0.22	0.06	0.14
Disease resistance	0.14	0.19	0.17	0.21	0.12	0.18	0.15	0.23	0.15	0.23	0.14	0.20
Insect resistance	0.09	0.15	0.12	0.17	0.16	0.18	0.17	0.23	0.12	0.17	0.23	0.23
Abiotic stress tolerance	0.08	0.15	0.10	0.18	0.09	0.17	0.10	0.17	0.10	0.19	0.07	0.16
Reduction in shattering	0.14	0.25	0.15	0.24	0.18	0.24	0.21	0.29	0.23	0.31	0.20	0.30
*Agronomic trait*												
Earliness	0.03	0.11	0.02	0.09	0.04	0.11	0.04	0.12	0.03	0.09	0.05	0.13
Intrahousehold decision-making power	0.51	0.22	0.49	0.22	1.00	0.00	0.53	0.17	0.47	0.17	1.00	0.00

Note: The VTIs are standardized to a value between 0% and 100% of the distance between the target and baseline VTIs.

### 5.2 Determinants of women's intrahousehold decision-making power

Table 7 shows the marginal effects of the fractional response model for WIDMP.^8^ The results suggest that wives' off-farm employment had a significant and positive effect on WIDMP, while the latter (and hence also MIDMP) was not significantly affected by husband's off-farm employment. If wives were engaged in income-generating activities outside of the farm, WIDMP increased by 8.0%.

**Table 7:** Results of the fractional response regression on women's intrahousehold decision-making power.

Variable	Marginal effect (SE)	
	Husband	Wife
Education	0.006 (0.008)	−0.012 (0.008)
Farming experience	0.002 (0.002)	−0.004 (0.002)**
Time preference	0.001 (0.002)	0.013 (0.007)*
Risk preference	0.003 (0.035)	0.054 (0.051)
Future perspective	0.060 (0.043)	0.089 (0.034)***
Off-farm employment	0.082 (0.060)	0.080 (0.041)**
Attendance to training	−0.042 (0.050)	−0.056 (0.048)
Membership to organization	−0.028 (0.037)	−0.001 (0.040)
		Household
Per capita income		0.000 (0.002)
Percent lease area		0.006 (0.037)
Proportion of production sold		−0.014 (0.063)
Buyer requirement		0.004 (0.042)
Credit		−0.077 (0.060)
Market information		−0.034 (0.037)
Climate change information		−0.005 (0.045)
Wet season		0.016 (0.025)

Notes: The coefficients are marginal effects after fractional response model estimation. The explanatory variables were tested for multicollinearity through the estimation of variance inflation factors (VIFs) and correlation coefficients (max VIF = 2.40; max correlation = 0.49).

N = 174, Log pseudo likelihood = −118.10.

Standard errors are clustered at the household level.

*, **, *** Indicates statistical significance at the 10%, 5%, and 1% levels, respectively.

Wives' time preference had a small, positive effect on WIDMP; a unit increase of their discount factor increased WIDMP by 1.3%. Wives' future perspective also had a significant effect on WIDMP. Those who claimed to account for future trends in farming conditions (market trends, climate change, etc.) in their optimal investment portfolios exhibited 8.9% higher WIDMP than those who based their portfolios on current and past problems.

Since age and farming experience were strongly correlated for both household members, we dropped the variable age in our model to focus on the variable that can somewhat be affected by intervention programs. Counterintuitively, we found that wives' farming experience had a small, negative effect on WIDMP in terms of investment decisions in future rice varieties, while husbands' farming experience did not significantly affect WIDMP (and hence MIDMP).^9^ Every additional year of wives' farming experience tended to erode WIDMP by 0.4%. Similarly, we did not find any evidence of a significant effect of education^10^, membership in organizations, attendance to trainings and our information treatments (Table 7); factors which are often claimed to increase women empowerment (Alkire et al., 2013; Alwang et al., 2017; Bertocchi et al., 2014; Carlsson et al., 2013; Meinzen-Dick et al., 2014; Orr et al., 2016).

## 6 Discussion and conclusions

In this study, we examined farm household decision making related to investment in plant breeding research. To do this, we conducted a framed field experiment with 122 rice farming households in Nueva Ecija, Philippines. Both the husbands and wives of these households were invited to participate in the experiment, and they were asked to indicate their preferences for investment in VTIs, relative to a preferred replacement variety, both as individuals and jointly as a household. We found that WIDMP is normally distributed and that, on average, women had almost equal (48%) decision-making power as men (52%) (Table 3, Table 6 and Fig. 2), revealing almost perfect gender equity in investment decision making in rice breeding. These findings are in line with The Global Gender Gap Report 2018 (World Economic Forum, 2018), which ranked (no. 8) the Philippines as one of the most gender-equal countries. It has closed about 80% of its overall gender gap in terms of four thematic dimensions: economic participation and opportunity, educational attainment, health and survival, and political empowerment.

We only found a few factors that can further improve WIDMP and the marginal effects are quite small. Remarkably, husbands' individual characteristics and attitudes did not significantly affect WIDMP and, hence, their own decision-making power in the household (MIDMP). The factors that had some marginal influence on WIDMP were women's off-farm employment, future perspective, time preference, and farming experience. Women were slightly more empowered in farm household decision making if they were engaged in off-farm employment and were less experienced in farming. Off-farm employment is likely to provide women with an opportunity to add to household income, gain knowledge and new information, which can increase her bargaining power in the household. This suggests that the expansion of women's portfolio of economic options outside the farm – rather than increasing their participation and experience in on-farm activities – may empower them in on-farm decision making (e.g., de Brauw, 2015; Twyman et al., 2015), in this case on investment in future rice varieties through rice breeding. This finding is consistent with the Sustainable Development Goals, which implicitly prioritize off-farm income generation over farm work in women empowerment.

More empowered women also tended to have a higher discount factor and base their investment decisions on anticipated future trends, rather than current or past experience like men (Table 3). This is somewhat consistent with women's farming experience being negatively correlated to WIDMP. Since the same variables of their husbands were non-significant (Table 7), this may suggest that wives' forward-looking arguments and perception of urgency tended to significantly persuade their husbands to converge in terms of VTI portfolio choice. These skills may have been developed because of her role in budgeting household expenses, or possibly as a result of involvement in economic and social activities outside of the farm (Eder, 2006).

Our finding that wives were able to participate, and influence household decision making and even had greater control over household income is consistent with recent work on women empowerment in four Southeast Asian countries. For example, Akter et al. (2017) found that in their Philippine study sites, decisions regarding rice farming are made jointly by husband and wife and that the wives have greater control over household income. Follow-up research is required to see whether our findings can be reproduced in other Southeast and South Asian countries (similar analyses are being conducted in Cambodia, India and Bangladesh).

Our study provides valuable insights on the importance of considering gender and intrahousehold dynamics when undertaking research on agricultural technology preferences and adoption decisions. Overall, the results of our study have implications in terms of the methodological approach in preference elicitation and variety development. As men and women have different preferences and roles within the household, it is important that methods for eliciting preferences should consider interviewing not only the household head, but also other household members involved in household and farm production decision-making processes. Following individual sex-disaggregated elicitation by joint elicitation has two benefits. First, it generates an additional and more robust round of consensus data, where both women and men have the chance to express their preferences. The results can then be translated into gender-responsive product profiles and incorporated into variety replacement programs and priority setting of breeding programs as well as extension and training programs. Secondly, it enables computing intrahousehold decision-making power as a proxy for women's empowerment and econometrically analysing the factors that affect gender influence on farm household decisions, in this case investment in portfolios of future rice VTIs to increase farms' performance and households' livelihood. The results can provide insights on the role of gender in formation of farm household preferences for technology attributes and can guide in targeting technologies (e.g., varieties) to particular segments of the farming population (e.g., farm households where women have limited outside options).

Our methodology further demonstrates that experimental approaches can be used to efficiently elicit intrahousehold decision-making behaviour, which can be captured, measured and analysed. In particular, participatory “gamification” approaches (e.g., Steinke and van Etten, 2017) combined with interactive tablet applications such as IGA are powerful methods for eliciting preferences and intrahousehold decision-making processes, while stimulating cognitive efforts of participants through a novel, exciting and engaging exercise.

The findings reveal broader implications in terms of women empowerment programs. Our evidence suggests that education and training programs alone could be insufficient for increasing women's bargaining power in the household; they need to be accompanied by investment programs generating off-farm employment opportunities for women in rural labour markets to absorb the skilled labour, as implied by the Sustainable Development Goals. Further research needs to be conducted, however, to validate our measure of WIDMP and assess how it correlates to other indicators of women empowerment in agriculture (e.g., WEAI – Women's Empowerment in Agriculture Index proposed by Alkire et al., 2013).

Our study, however, has limitations regarding its sample selection of rice farmers in a single region of the Philippines. This means that generalization of the findings to a larger population is also limited. Specifically, variety choice is site-specific and it is recommended that similar research is done in other major rice-growing areas to help in the development of rice variety product profiles that better target their preferences and needs. Furthermore, this research could be extended to consider other agricultural commodity sectors where significant public dollars are invested globally in research and development of new crop varieties.

## Footnotes

1 Farmer participants in different sessions did not have the opportunity to discuss the IGA as the sessions were conducted in the village (in a village hall), with a two-hour interval between the morning and afternoon sessions to avoid any interactions among participants from different sessions. Participants were not informed of the other villages selected for the experiment.

2 At the time of the experiment (February 2016), one US dollar was equivalent to approximately PHP 48.

3 During the experimental sessions, a considerable amount of time (1 h) was spent on presenting and explaining each trait and the improvements that could be achieved. Visual stimuli and/or analogies were used in the explanation of traits (see Appendix A).

4 Metro Manila is a major market for the rice produced in Nueva Ecija. Given the trends in consumption patterns and urbanization, this major market can provide farmers opportunities to increase their incomes if they will be able to provide the grain quality traits demanded by the consumers.

5 We did not specify how long the participants should aim to finish the IGA. However, we found that it took on average 5 min for the participants to complete one round of IGA.

6 At the time of experiment, the typical daily wage rate for agricultural labour in the province was around PHP 334 (Philippine Statistics Authority, 2016b).

7 Pre-testing of the IGA experiment was conducted in Victoria, Laguna, Philippines in May 2015. The session took 2 h to finish (Demont et al., 2015). The IGA experiment was then conducted in Eastern India in October 2015, in Bangladesh in September–October 2016 (Ynion et al., 2015, 2016) and in Cambodia in September 2018 (IRRI, 2018).

8 In the final analysis, n = 174. One case was dropped as the portfolio of VTIs chosen by the wife and husband individually exactly matched the portfolio they selected jointly. In this case, intrahousehold decision-making power cannot be measured through VTI portfolio choices.

9 A reviewer pointed out that there might be correlation between farm experience and off-farm employment. However, the correlation coefficients between farm experience and off-farm employment were low: 0.14 (husband) and 0.08 (wife). The variance inflation factors (VIFs) were 2.40 (husband) and 1.84 (wife) for farm experience and 1.46 (husband) and 1.49 (wife) for off-farm employment.

10 Contrary to expectations, a wife's education had a negative, but non-significant, effect on WIDMP (see Table 7). A reviewer pointed out that this might be due to potential correlation between a wife's education and off-farm employment. This is likely not the case as the correlation coefficient (0.26) and VIFs (education = 1.64; off-farm employment = 1.49) are rather low.
