# Supplementary material for: Off-farm employment increases women's empowerment: Evidence from rice farms in the Philippines
Source: J Rural Stud. 2019 Oct;71:62–72. doi: 10.1016/j.jrurstud.2019.09.002 (PMC6876659; doi:10.1016/j.jrurstud.2019.09.002)
Supplement: Multimedia component 2 [file mmc2.docx]

**Off-farm employment increases women’s empowerment: Evidence from rice farms in the Philippines**

**Appendix A. Supplementary Material**

**Experimental Protocol for the Investment Game Application (IGA)**

Rio Maligalig^a^, Matty Demont^b^, Wendy J. Umberger^a^, Alexandra Peralta^a^

^a^ Centre for Global Food and Resources, Faculty of the Professions, The University of Adelaide, Level 6 NEXUS 10 Tower, 10 Pulteney Street, SA 5005, Australia

^b^ Agri-food Policy Platform, International Rice Research Institute (IRRI)

DAPO Box 7777, Metro Manila 1301, Philippines

# Technical Details for the Investment Game Application (IGA)

The Investment Game Application (IGA) features three interconnected models: (i) a flexible fixed-cost quadratic (FFCQ) cost function, (ii) a risk function, and (iii) a stochastic return function. These functions were estimated through an expert elicitation workshop with rice breeders from the International Rice Research Institution (IRRI) and National Agricultural Research Systems (NARS) partners (Demont and Villanueva, 2019). The IGA features 11 traits (four grain quality traits, i.e. slenderness, aroma, (un)stickiness, and head rice recovery; five stress tolerance traits, i.e. lodging tolerance, disease resistance, insect resistance, abiotic stress tolerance, and reduction in shattering; an agronomic trait, i.e. earliness; and a by-product trait, i.e. straw digestibility). Traits featured in IGA can be omitted or modified according to the context. For example, in South Asia, consumers tend to prefer loose, unsticky rice, while in Southeast Asia, sticky rice is preferred (Custodio et al., 2016). Therefore, IGA can be adapted to feature “unstickiness” in South Asia and “stickiness” in Southeast Asia. In the case of the Philippines, a reduced IGA with 10 traits is used, i.e. straw digestibility is omitted as this trait is less relevant in this context.

To generate data for the FFCQ cost function, for each of the 11 traits breeders first defined a baseline and target trait value (Table 1 in paper) that could be achieved with a US$1 million breeding grant, and then estimated: (i) the fixed costs that would be required to start up the breeding program; (ii) the total costs to achieve the target value; and (iii) the total costs that would be incurred to achieve half of the target value. For each of the 55 trait combinations (pairs), breeders then estimated the fixed and total costs, taking into account economies of scale and scope.

A FFCQ with 89 parameters (an intercept representing the overall overhead cost, 11 fixed costs, 11 coefficients for the linear terms, 11 coefficients for the quadratic terms, and 55 coefficients for the interaction terms) was then fitted on the data and incorporated in the IGA. The FFCQ simulates the breeding costs involved in improving the varietal trait improvement (VTI) of each trait from the baseline up to the target level and all combinations of VTIs selected in the investment portfolio (Demont and Villanueva, 2019). The FFCQ function translates the portfolio of VTIs into a cost, which is deducted in real time from the budget bar. The budget constraint ensures that farmers do not propose unrealistic portfolios of VTIs and compels them to prioritize between alternative VTIs.

Due to the flexible fixed-cost nature of the cost function, initial improvements of a trait (from the baseline to the first increment of 5% in the VTI) generate higher costs than further incremental improvements due to fixed start-up costs such as the establishment of new laboratory or field experiments. Further improvements induce variable costs that exponentially increase over increasing VTI levels due to the convex nature of the FFCQ cost function. Economies of scope and scale in rice breeding are captured by the cost function as well; most portfolios of VTI pairs are sub-additive, while some are super-additive, e.g. the combination of slenderness and head rice recovery (Demont and Villanueva, 2019).

For each of the 11 traits at both the VTI’s target and mid-target values, breeders also estimated: (i) the probability of success of achieving the VTI; and (ii) the social returns the VTI would generate due to yield or price increase. We then estimated the risk and return functions by fitting log-linear curves running through both points and the origin, assuming that zero investment would generate a deterministic zero return. These functions were incorporated into the IGA. Under the VTI bars, a pie diagram indicates the probability of success of the rice breeding program in achieving each chosen VTI level. This helps farmers to match their VTI preferences with their investment risk preferences. The return function finally generates a random draw of the stochastic social returns a particular portfolio of VTIs could generate, taking into account the risk levels of each VTI.

# Ethics Approval

The University of Adelaide’s Human Research Ethics Committee approved the experimental procedures and all data collection instruments (Ethics Approval Number H-2016-010). Written informed consent was obtained from all individual participants prior to the actual experiment.

# IGA Experimental Procedures

The following section provides the instructions and script (in *italics*) that the facilitator and research team followed during the experimental sessions. The sessions were conducted using the local language (Filipino) and were accompanied by a visual presentation on a large screen.

Each session started with the facilitator explaining the consent form which was provided to each participant. The consent form explained to the farmer-participants that their participation was completely voluntary and that any information they provided would only be used for research purposes. We also asked for their permission through the form to allow the team to take videos and photographs during the experiment. Participants were then given time to read the form and were asked to sign if they agreed to the conditions outlined in the consent form.

After obtaining the participants’ consent, a trained facilitator introduced the members of the IGA research team to the participants and expressed their gratitude to them for accepting the invitation to participate in the activity. Afterwards, the information treatment assigned for the session was introduced. Participants in each session were randomly assigned to a particular information treatment – market trends^[[1]](#footnote-1)^, climate change^[[2]](#footnote-2)^, market and climate change, and a control. Subsequently, the purpose of the IGA experiment was explained and active participation was motivated by pointing out to the farmers that their participation and expert knowledge were very significant for the future success of breeding “ideal” rice varieties that correspond to their needs and that their decisions could lead to improvements in their livelihoods.

## Introduction

**Greetings**: *Good morning/afternoon to all of you. First, we would like to thank all of you for accepting our invitation to participate in this activity, which will provide breeders feedback on farmers’ preferences for rice varietal traits.*

**Introduce facilitator and the research team members**: *I am* [insert name of facilitator, designation, organization]*. I will be the one who will explain our activity for today. Also, here with us are* [insert name of agents/enumerators] *who will help and guide you during the activity.*

**Introduce IRRI and The University of Adelaide research team:** *This research is being conducted by IRRI and The University of Adelaide. Researchers from these institutions are involved in the design and implementation of this study and in the data analysis.*

**Introduce the activity for the day:** *Our activity for today involves two parts. In the first part, you will be asked to perform a task, where our agents will be with you to assist you. This task will take about two hours – from the explanation of the research and instructions to the actual implementation of the task. In the second part, you will answer a short paper-based survey questionnaire, which will also be administered by our agents. This will only take about 45 minutes. You can take a break after completing the task and before you start the survey. We will provide some light snacks during your break. If in case you feel discomfort or tiredness during the conduct of the task or the survey, please let the agents know. We will now proceed with the explanation of the task (Investment Games).*

## Instructions and information

### Introduction

#### The following text in italics was used to explain the purpose of the activity. A summary of the objective of each explanation is provided before the script.

**a.** **Explain the International Rice Research Institute’s (IRRI) mission through rice breeding programs funded by donors:** *The primary mission of IRRI is to reduce poverty and help farmers improve their livelihood. IRRI receives funds from donors to perform different activities which help to achieve these goals. One of them is the development of new rice varieties through IRRI’s breeding programs. We want to involve farmers more actively in breeders’ decisions on which traits of rice varieties need to be improved and prioritized. With this new strategy, we hope to tailor the varieties better to farmers and consumers’ needs and foster adoption rates.*

*In this activity, you will be involved in a new approach to public investment in rice breeding. As you know, donors invest funds in IRRI and NARS (National Agricultural Research Systems) breeding programs (e.g., PhilRice) to help these programs in the development of new varieties for farmers. Today, we will give you the opportunity to invest in rice breeding programs based on the traits of the varieties you prefer to be improved. Funds from donors will be distributed to the farmers, who can now decide on their best use for developing the ideal rice varieties they always wanted to have. We believe that farmers are the experts in identifying how their varieties can be improved to increase their livelihood.*

*To determine the traits of the varieties that farmers want to improve and at what extent of improvement, we will use a tablet application called the Investment Game Application (IGA). This application will help breeders in prioritizing the improvement of rice characteristics farmers want in order to obtain their ideal rice varieties.*

**b.** **Outline the opportunity for farmer-participants to become shareholders in the rice breeding programs:** *Today, we will create a real, but temporary market for investment in public rice breeding. Moreover, we endow you with an investment share in real money. By creating your ideal variety, you are in fact investing this money in public rice breeding. This will allow you to gain a real return to your investment, which you can take home. In other words, today you will be an investor of the rice breeding program and decide on how your share should be invested in the development of rice varieties which are suitable to your needs.*

#### **Motivate farmer-participants on the importance of their active participation in this activity:** *We know that IRRI and NARS breeders produce new rice varieties for the benefit of farmers and consumers. Therefore, it is important to involve the expert knowledge of farmers on the variety characteristics they want to improve before allocating resources within the breeding program. This activity provides you with the opportunity to provide breeders with feedback on your preferences for rice varietal traits. We kindly request for your participation in this activity.*

### Training on the Investment Game Application (TIGA)

*Before we proceed to the actual task (investment game), let us have a short training first.*

#### **Explain the overall goal of the investment game:** *The goal of the game is to compose the ideal rice meal by finding out your preference trade-offs for viands*.

#### **Provide instructions to participants on how to play the game:** *It is lunchtime and as you are passing by a restaurant, you saw a promotional offer of free rice for a meal with two choices: vegetable dish (pakbet) and meat dish (adobo). Reaching your pocket, you found that you have only 50 PHP allotted for your lunch meal. What proportion of your budget will you allocate for vegetable and for meat? Just like in a popular product commercial: “Up to what extent your 50 PHP can buy.”*

The facilitator showed the interface of the Training Investment Game Application (TIGA). He/she oriented the farmers on its components and features (the plates for vegetables and meat dish, the spin button and the budget bar) and how to use the TIGA).


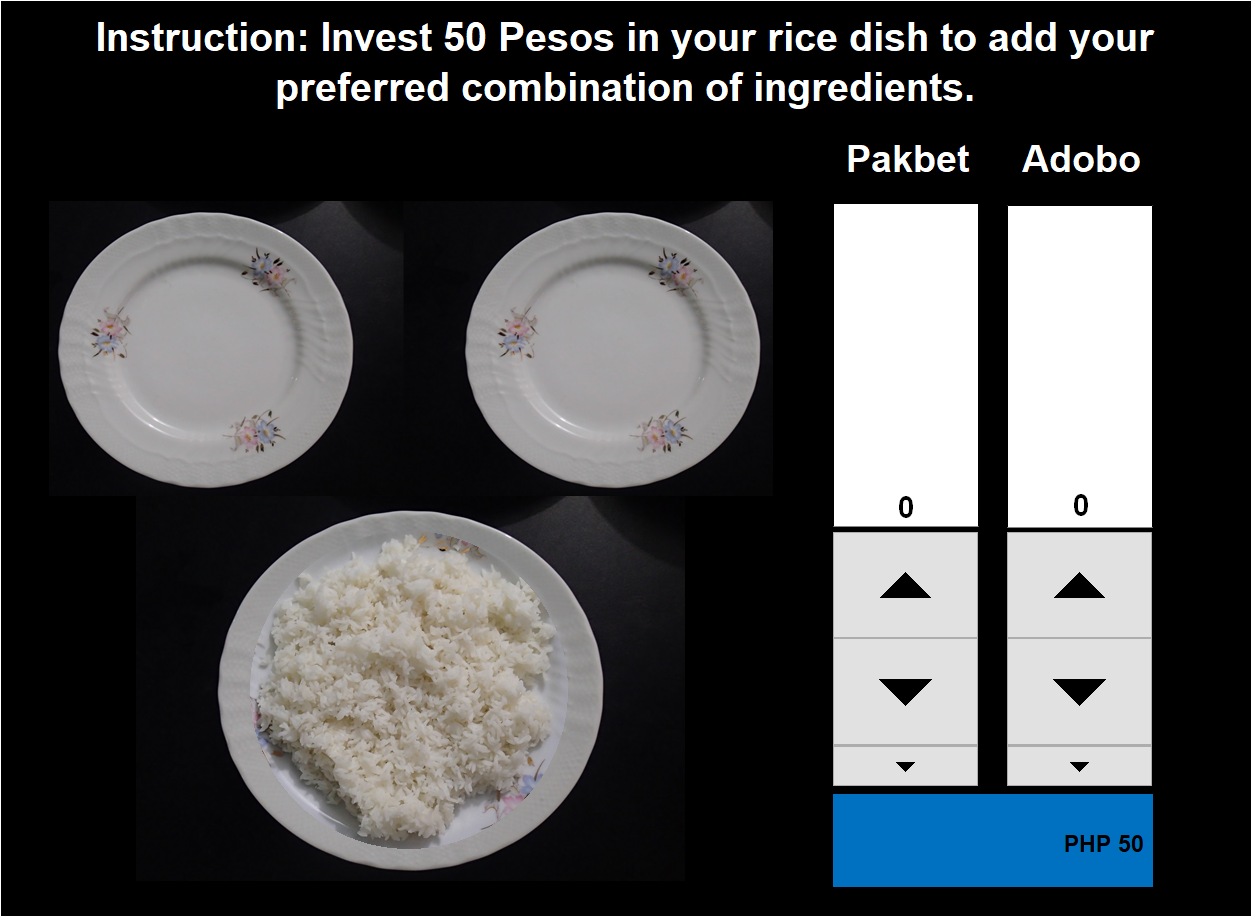


Figure 1. TIGA interface.

#### **Hands-on training**

The participant completed the training exercise (TIGA) through the guidance of assigned agents.

### Introduction of Investment Game Application (IGA)

*Did you enjoy our short exercise? How was your experience playing with the tablet application? We will do this again later but will use the “investment game application” (IGA) applied to rice varietal improvement. The IGA has the following parts:*

#### **Explain “Rice Varietal Traits” and their logo:** *This part shows the rice varietal traits and their corresponding logo.*


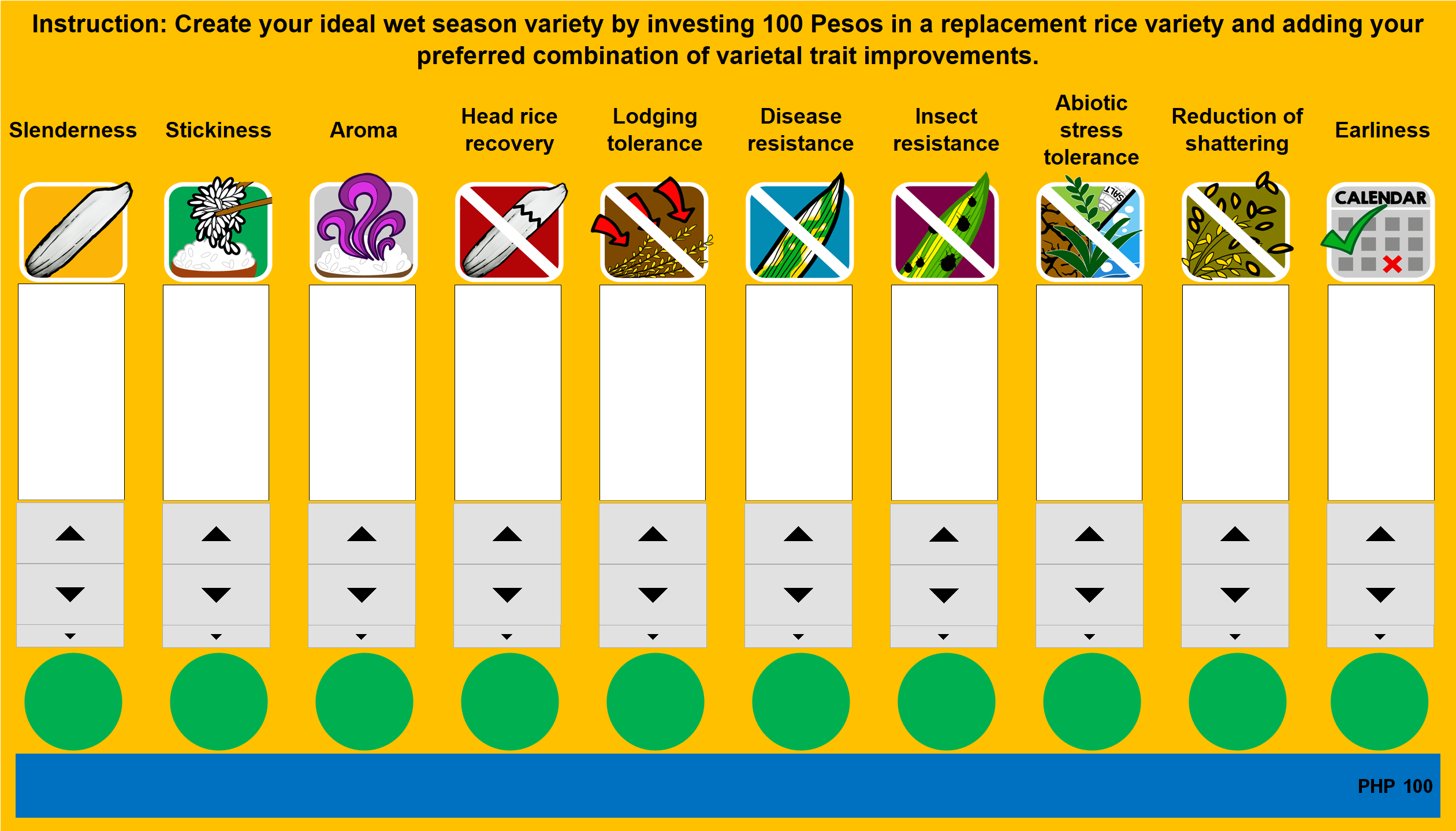


Figure 2. IGA interface highlighting the rice varietal traits and logo.

#### **Explain “Varietal Trait Improvements” and the “Spin Button”:** *This portion displays the level of varietal improvement of each trait and the spin button below is used to increase and decrease the improvement level.*


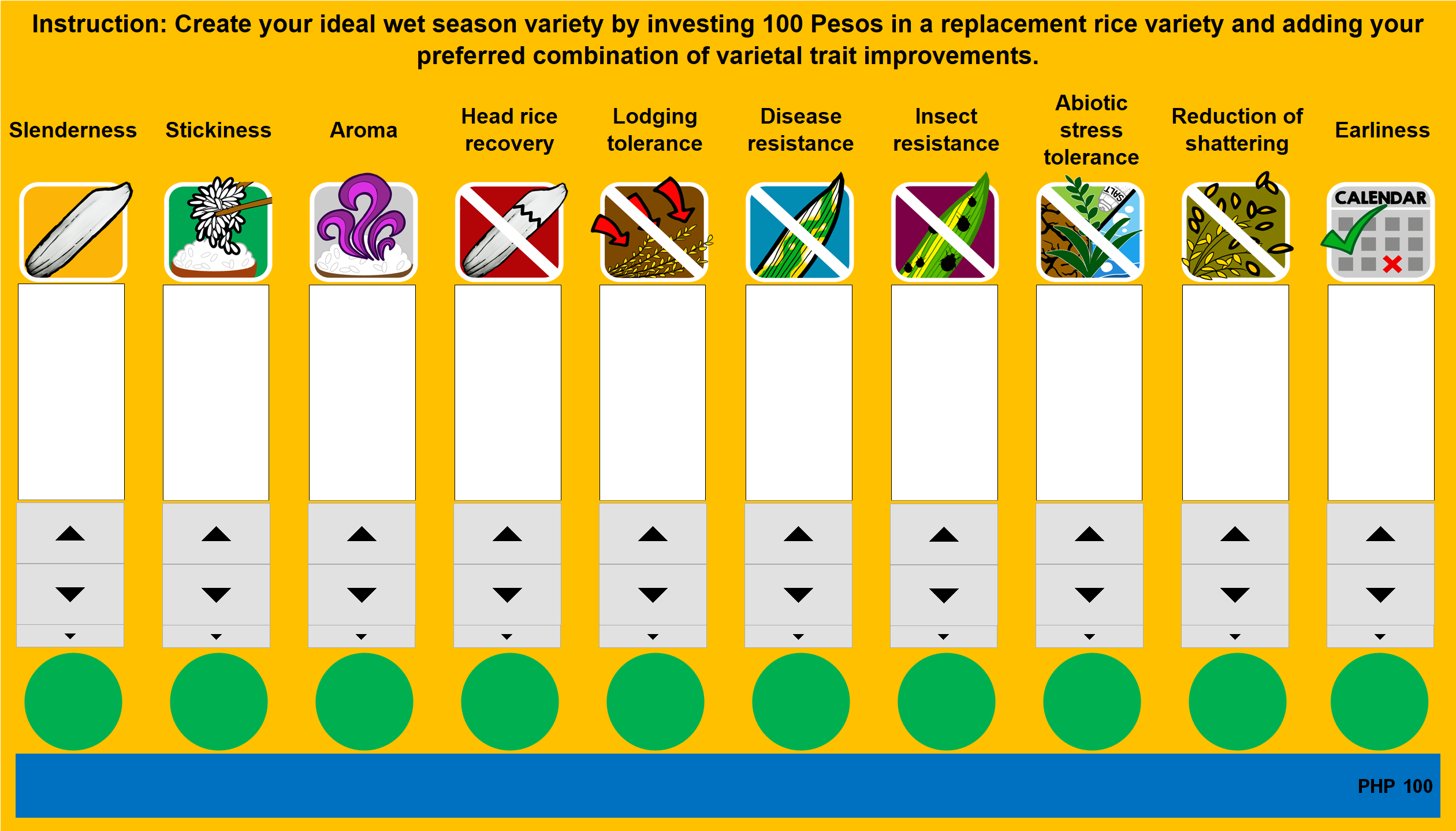


Figure 3. IGA interface showing (in red box) the VTI bars and spin button.

#### **Explain “Investment Risk” (e.g. lottery):** *The pie chart located at the bottom of each trait corresponds to the investment risk, which is based on the level of varietal improvement. Green color indicates the chance or probability that the breeding program will be successful in developing a variety with exactly your required level of trait improvement. Whereas, red color indicates the chance or probability that the variety, which will be developed, will have lesser expected level of trait improvement, i.e. somewhere between your required level and zero. The larger the green area, the higher the chance of getting exactly your required level of trait improvement and the bigger the red area, the higher the chance of getting lesser expected level of trait improvement, i.e. somewhere between your required level and zero.*


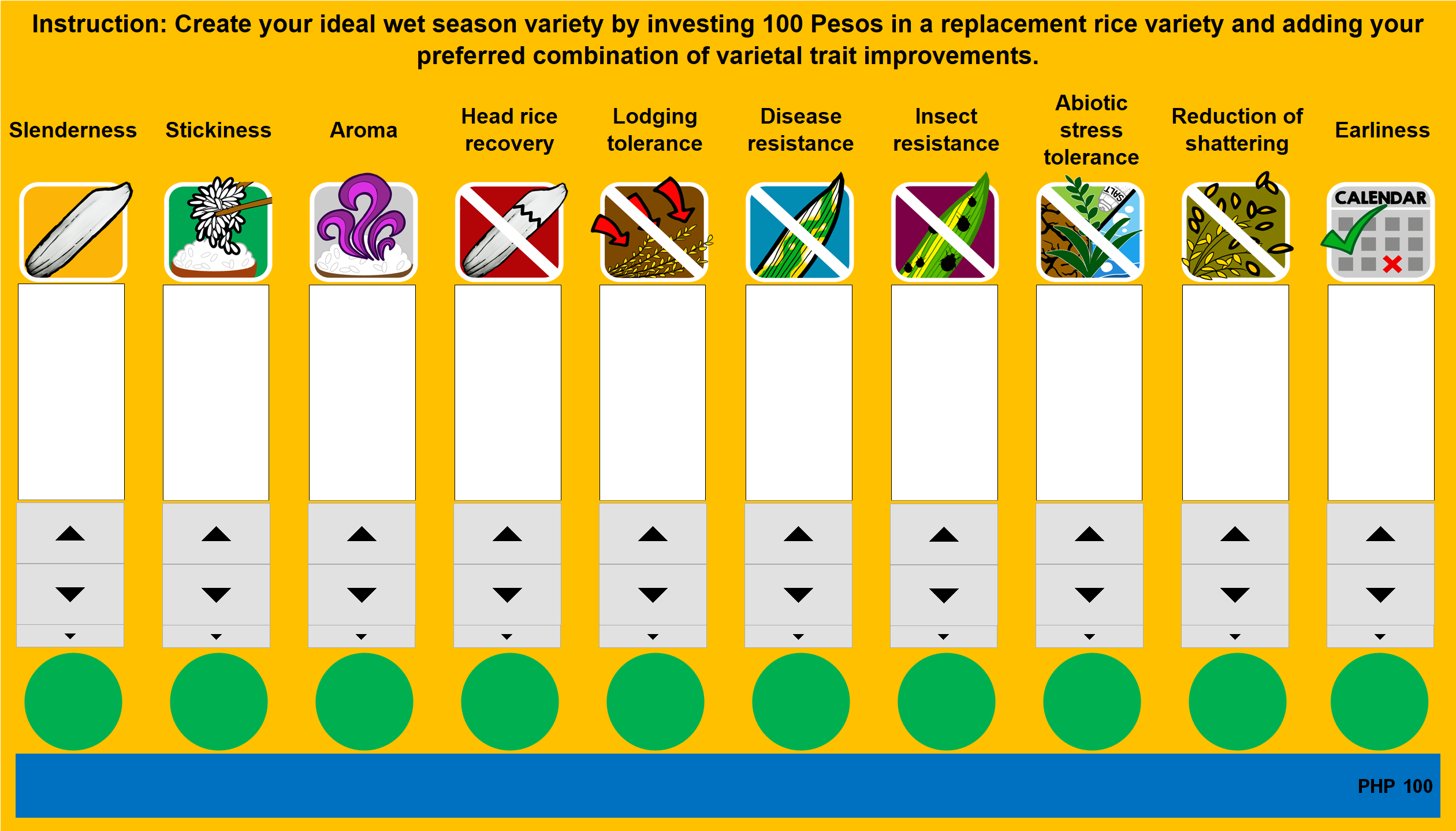


Figure 4. IGA interface showing the pie charts (in green) corresponding to investment risk.

#### **Explain the “budget bar”:** *This bar shows your budget status. Blue color indicates the balance of your fund. As you invest in a single trait or multiple traits, there will be a corresponding reduction to your budget.*


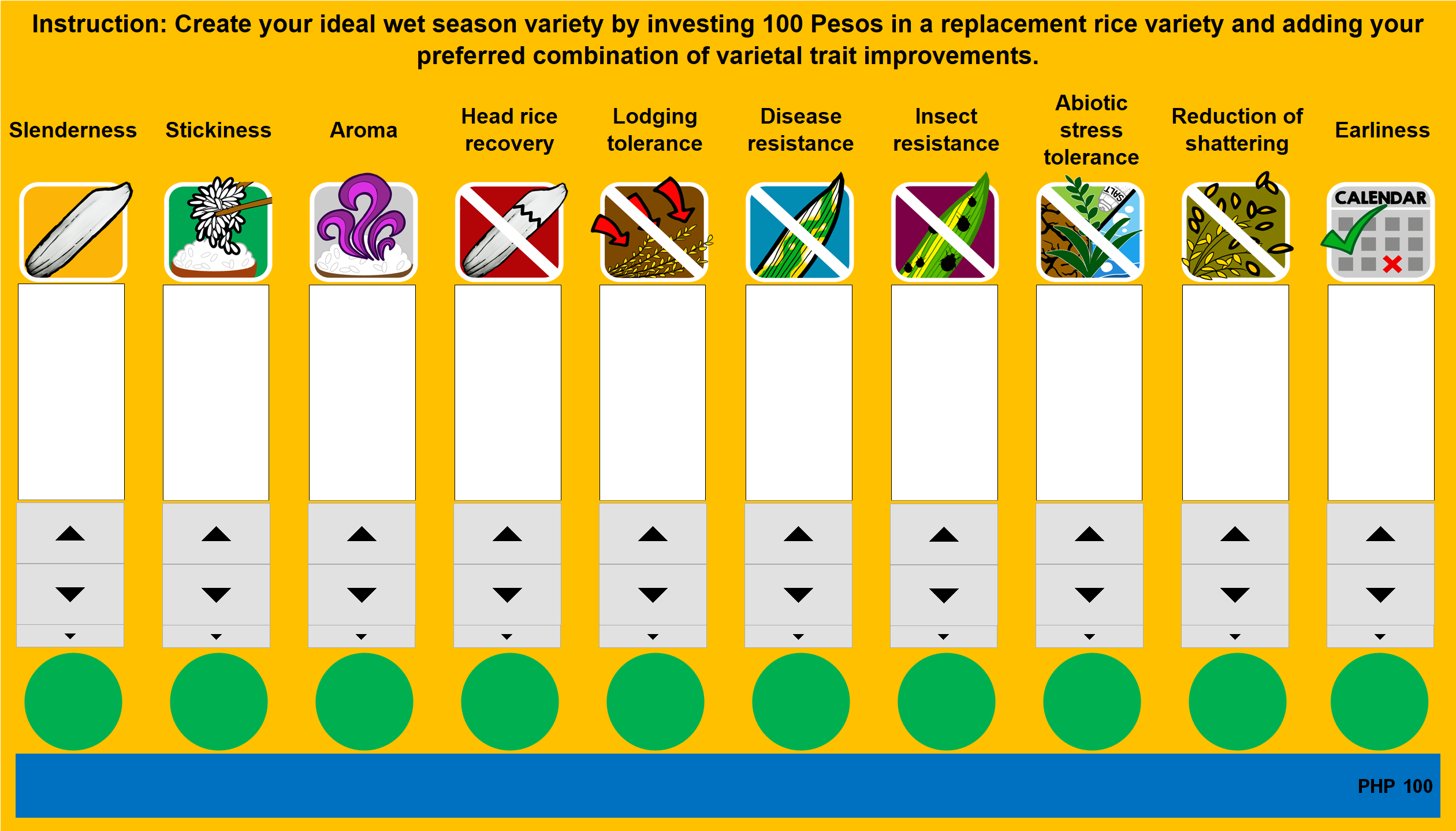


Figure 5. IGA interface showing the budget bar (in blue).

#### **Explain the “replacement rice variety”:** *When thinking about the trait improvement, it is important to refer to a “replacement rice variety”. This variety can be your most preferred or a popular variety. You may or may have not grown it in the past or currently growing. What is important is that you are familiar with its characteristics and it is the variety that you would like to improve for selling.*

### Description of rice varietal traits and target on trait improvement

*What are the rice traits you want to add or improve in your replacement variety? According to rice breeders, they can improve 10 rice traits up to a certain point in a span of six years. Let me first discuss each rice trait.*

#### **Explain “Slenderness” (in local language, in PH: Pagkapayat na hugis):** *This trait is about the shape of rice grains. Shape of a grain refers to its length and width. Some rice varieties are long and slender, some are short and bold. Looking at the image* [referring to the slide presentation of baseline]*, if your replacement rice variety, which is mainly for selling, has this length and width (i.e. medium) and you want to make it slender, you can choose this trait and improve it by pressing the upper spin button up to the level of slenderness you want.*

| 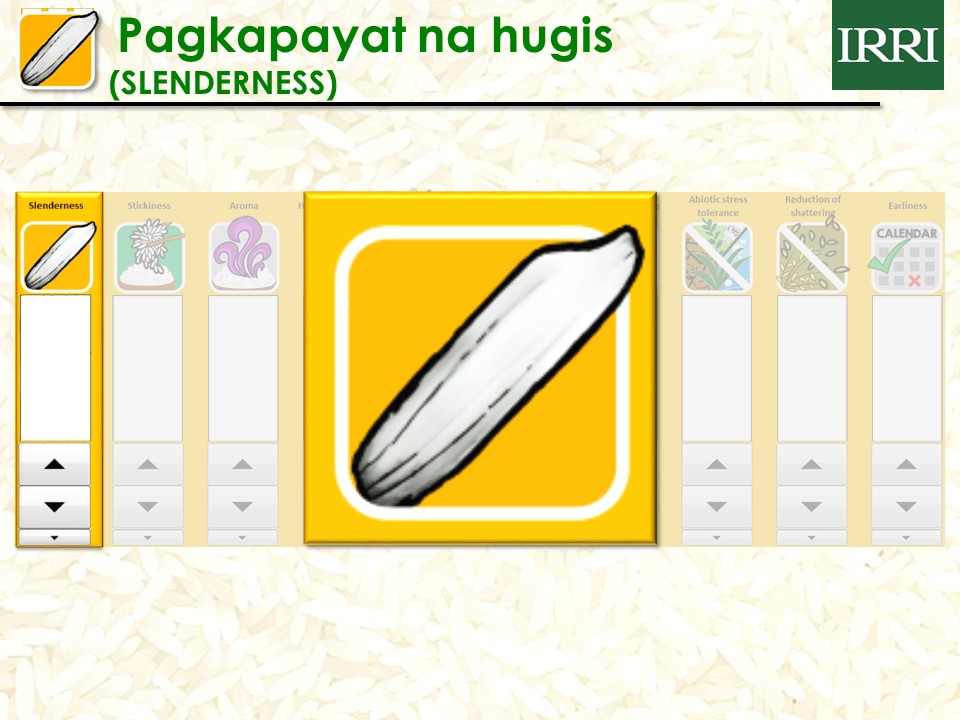(1) | 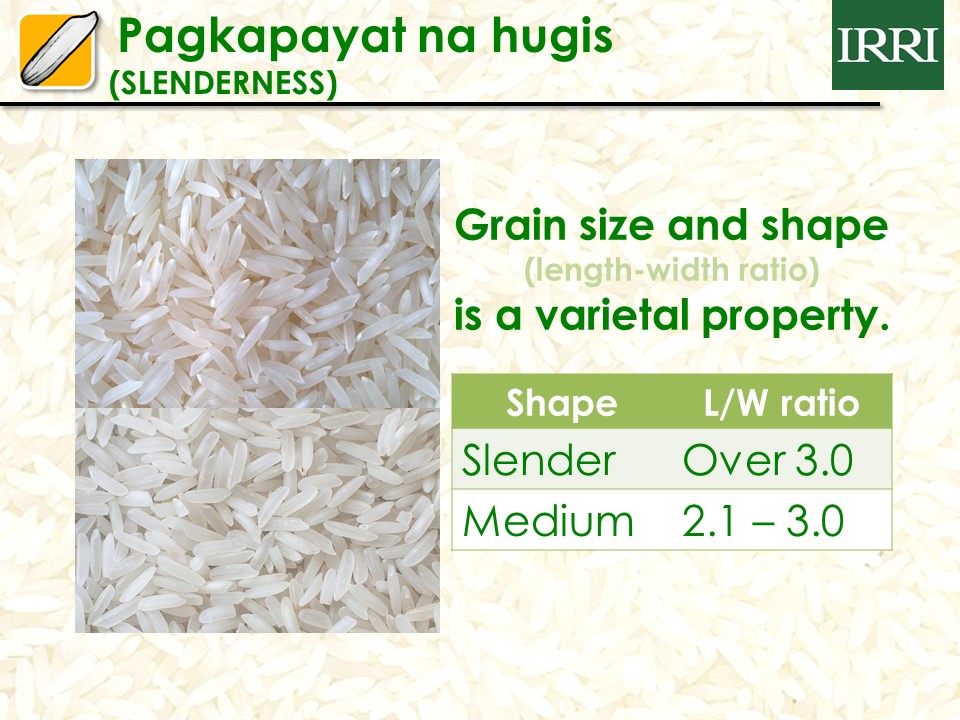(2) |
| --- | --- |
| 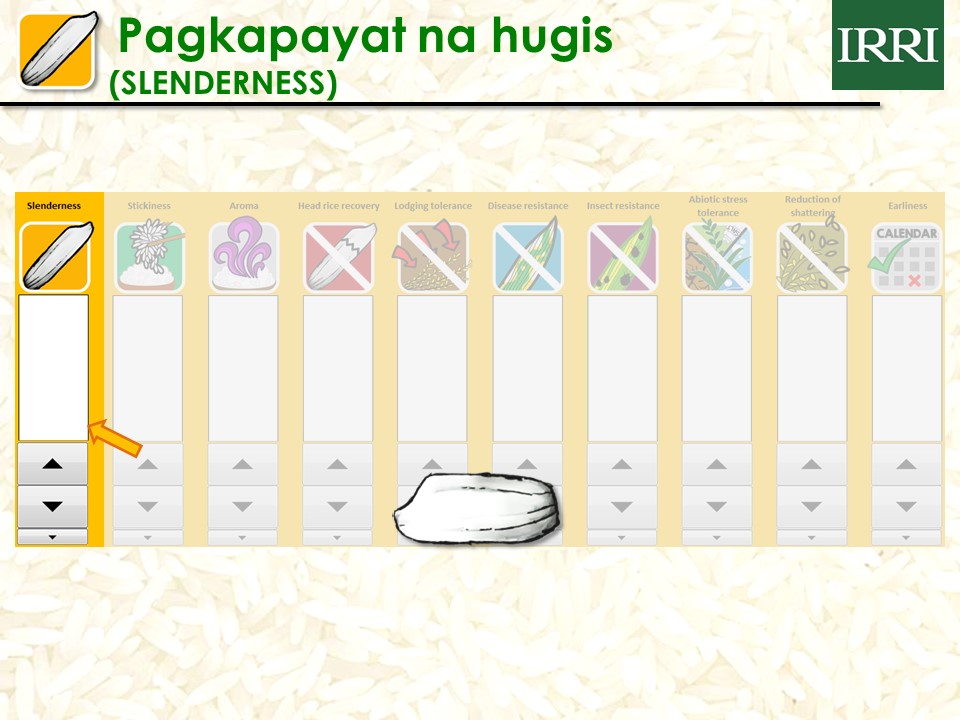(3) | 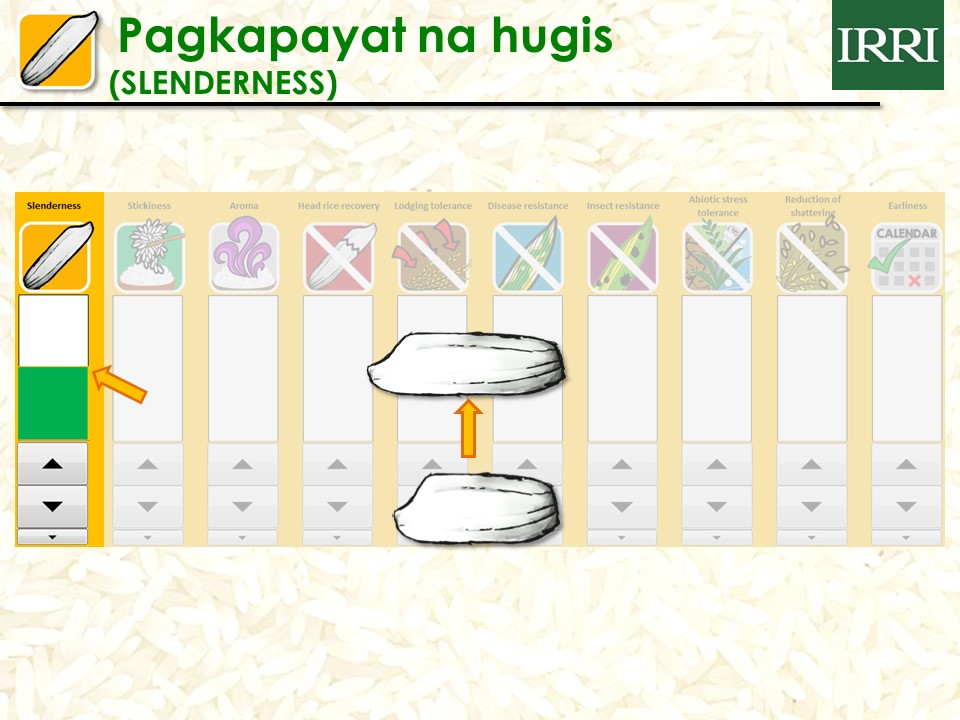(4) |
| 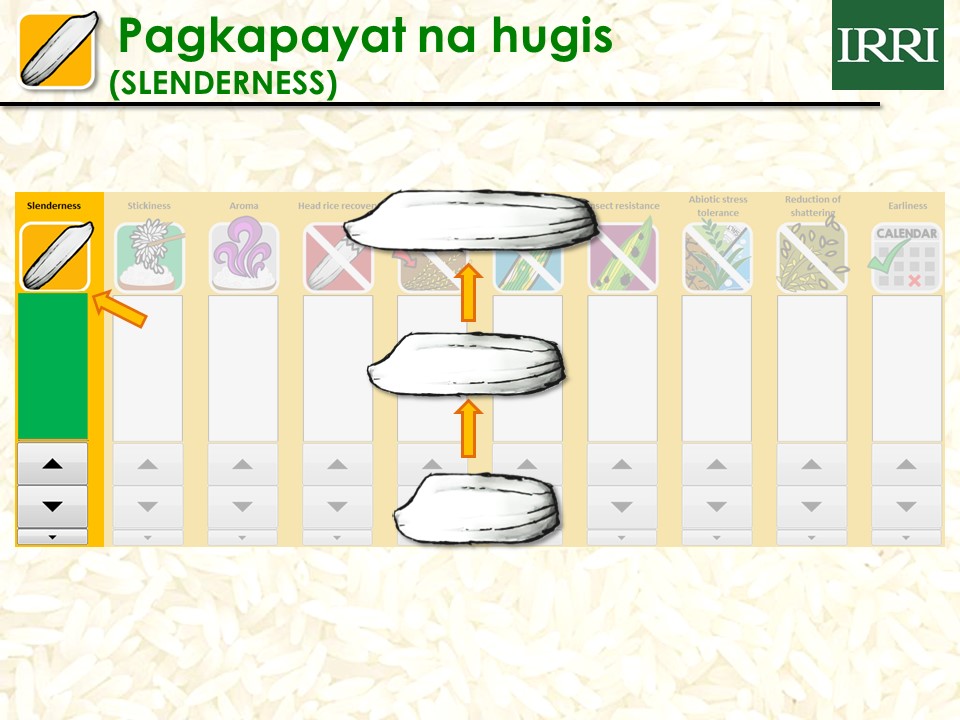(5) |  |

Figure 6. Slides used to explain slenderness and its VTI levels.

#### **Explain “Stickiness” (in local language, in PH: May kalagkitan):** *There are rice varieties that are soft and sticky while other varieties are hard and dry. If you want your replacement rice variety, which is mainly for selling, to have a softer and stickier texture, kindly choose this trait and increase the level of stickiness up to your desired level. For example, if your replacement variety is hard and dry and you want to make it softer and stickier, increase the level up to your desired level.*

| 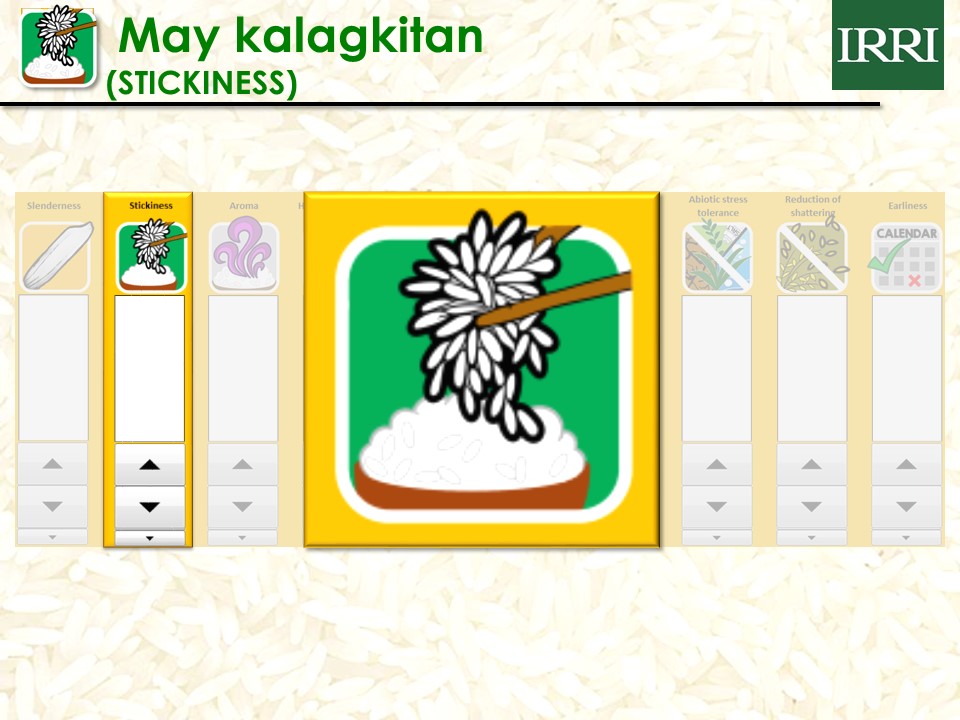(1) | 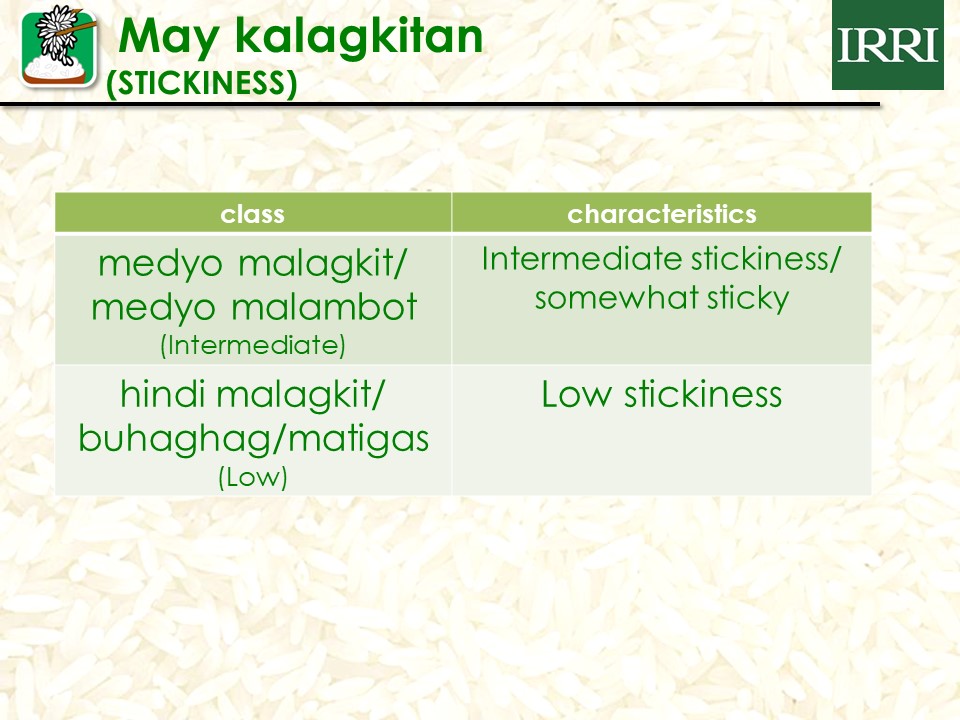(2) |
| --- | --- |
| 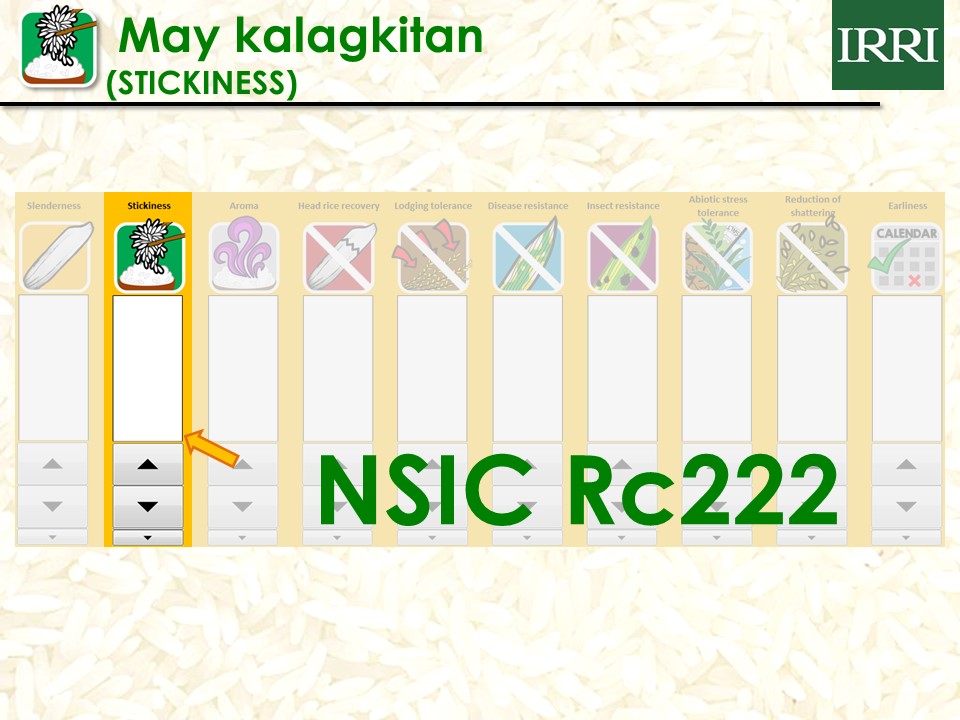(3) | 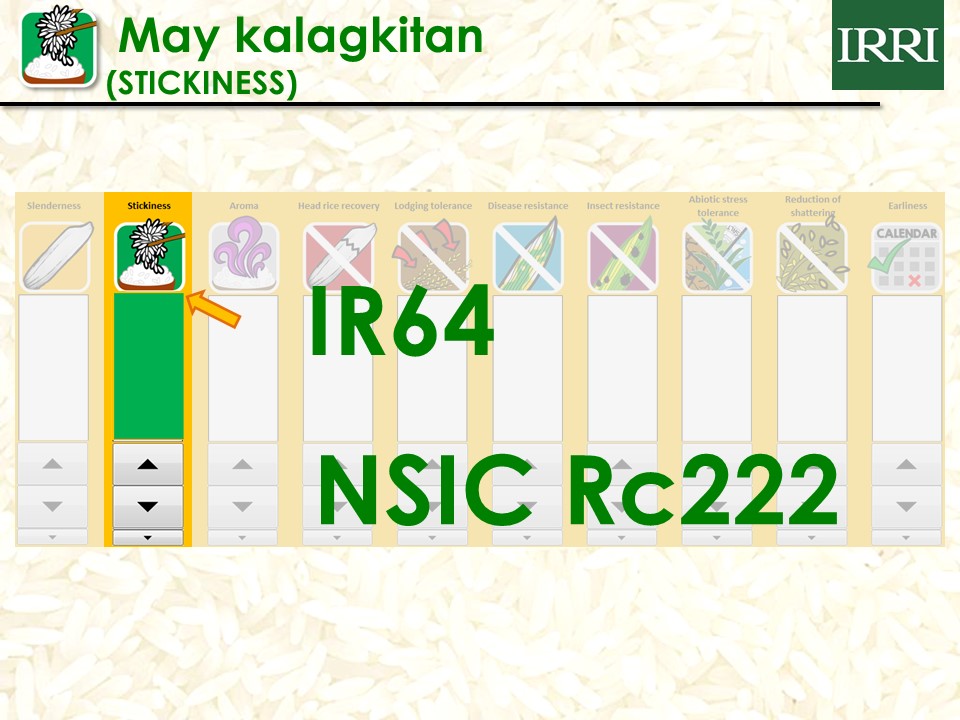(4) |

Figure 7. Slides used to explain stickiness and its VTI levels.

#### **Explain “Aroma” (in local language, in PH: Bango):** *Here in the Philippines, we can say that rice has a good aroma if it has attractive fragrance and smells like “pandan” leaves. The most popular aromatic rice in Philippine market is Jasmine rice. In South Asia, Basmati is one of the popular aromatic varieties. If you want to improve the fragrance of your replacement variety, which is mainly for selling, or eventually transform it into aromatic rice, choose and invest in this varietal trait.*

| 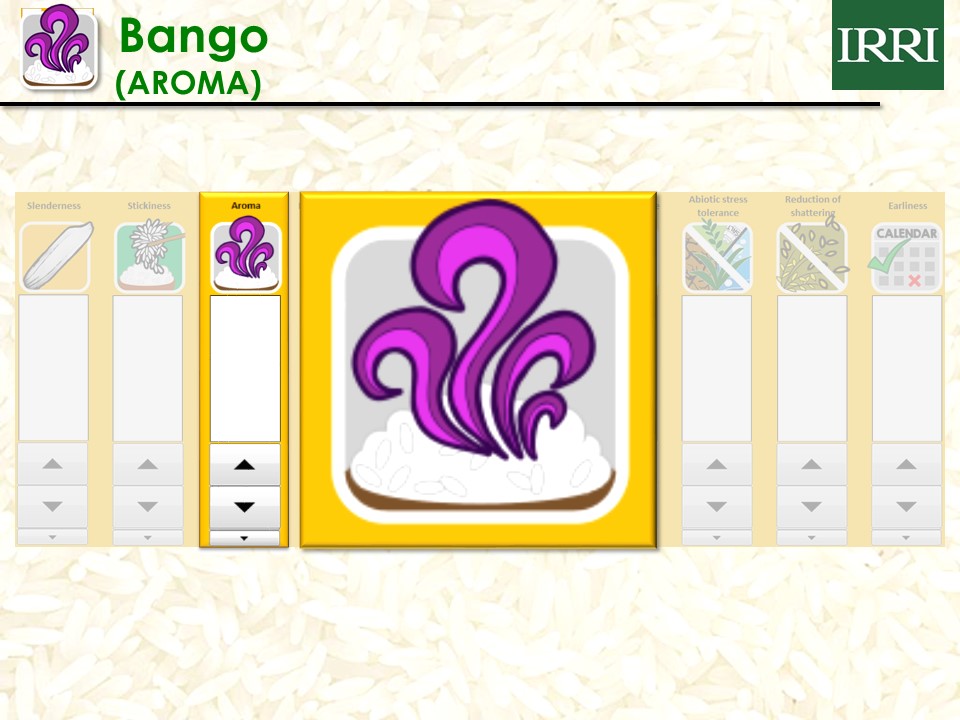(1) | 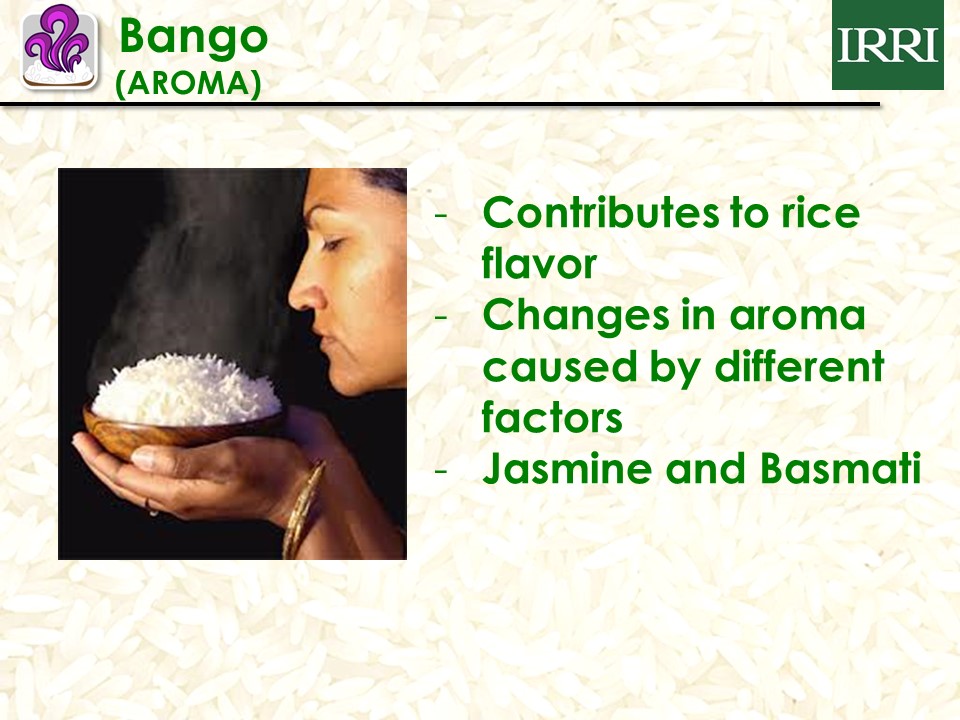(2) |
| --- | --- |
| 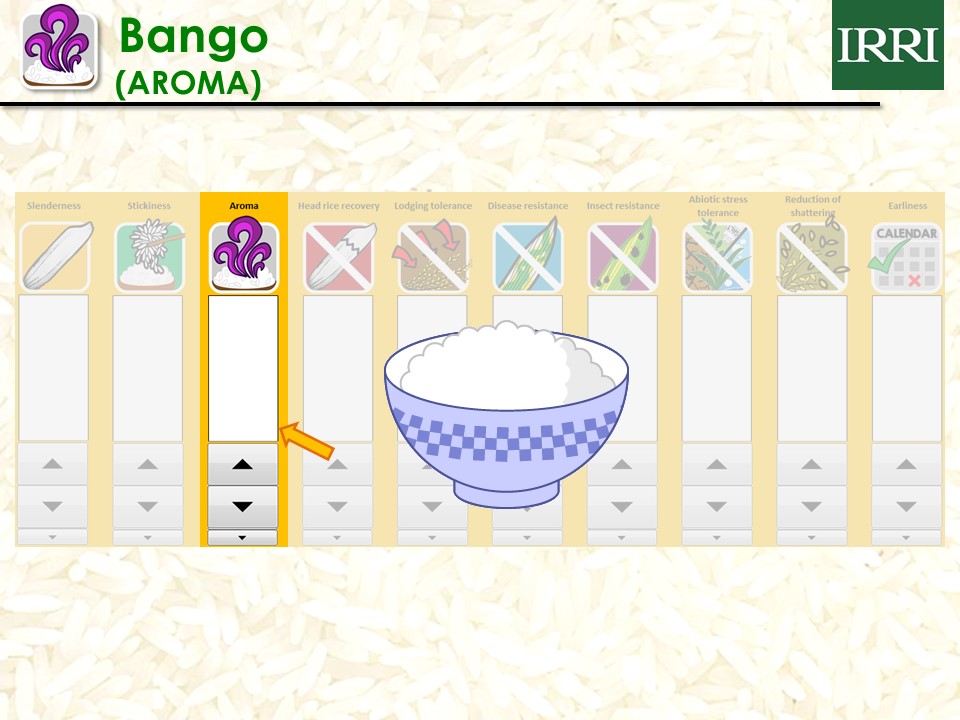(3) | 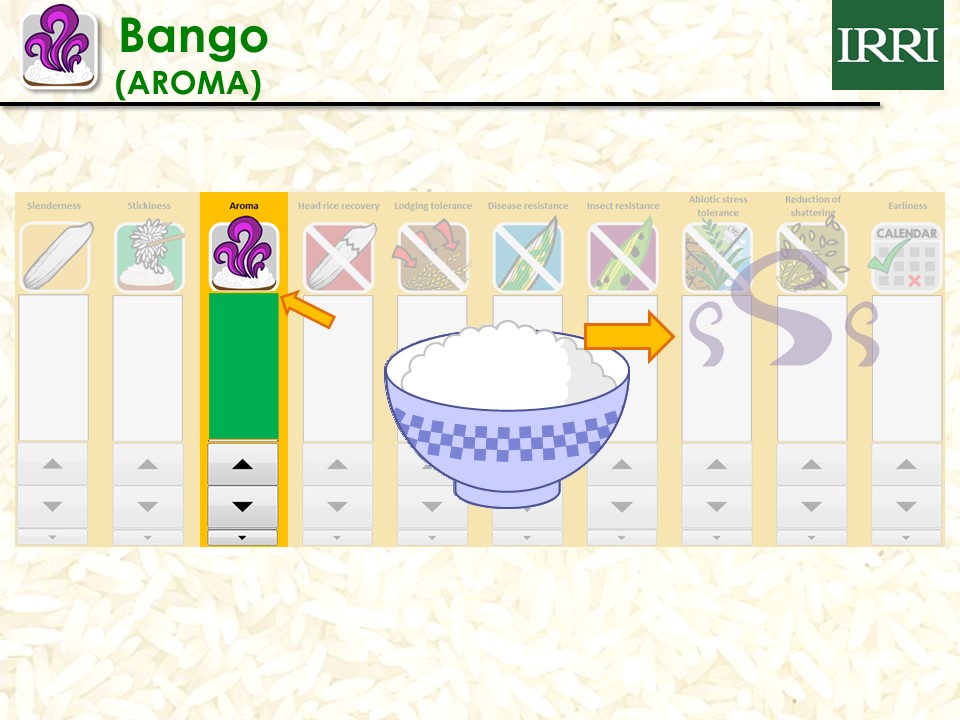(4) |

Figure 8. Slides used to explain aroma and its VTI levels.

#### **Explain “Head Rice Recovery” (in local language, in PH: Pagkabuo matapos gilingin):** *This trait refers to the amount of recovered whole grain against broken grains after milling. Head rice recovery has three categories: Grade 2 (39.0%–47.9%), Grade 1 (48.0%–56.9%) and Premium (57% and above). If your replacement variety, which is mainly for selling, has around 45% head rice recovery (Grade 2), pulling up the bar up to its maximum can improve its head rice recovery up to 60% (Premium).*

| 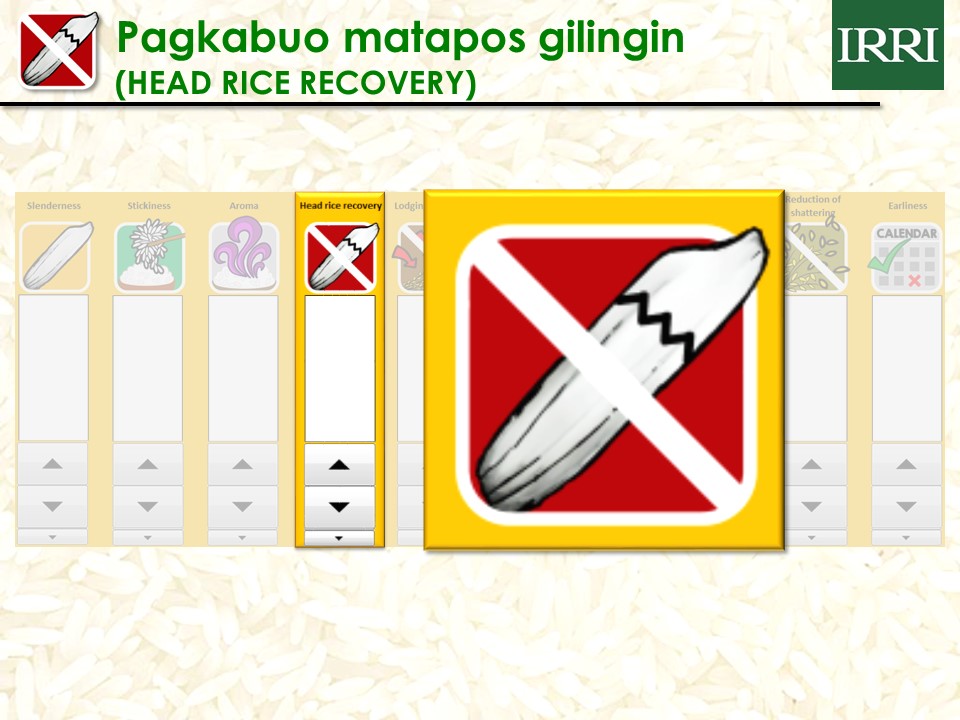(1) | 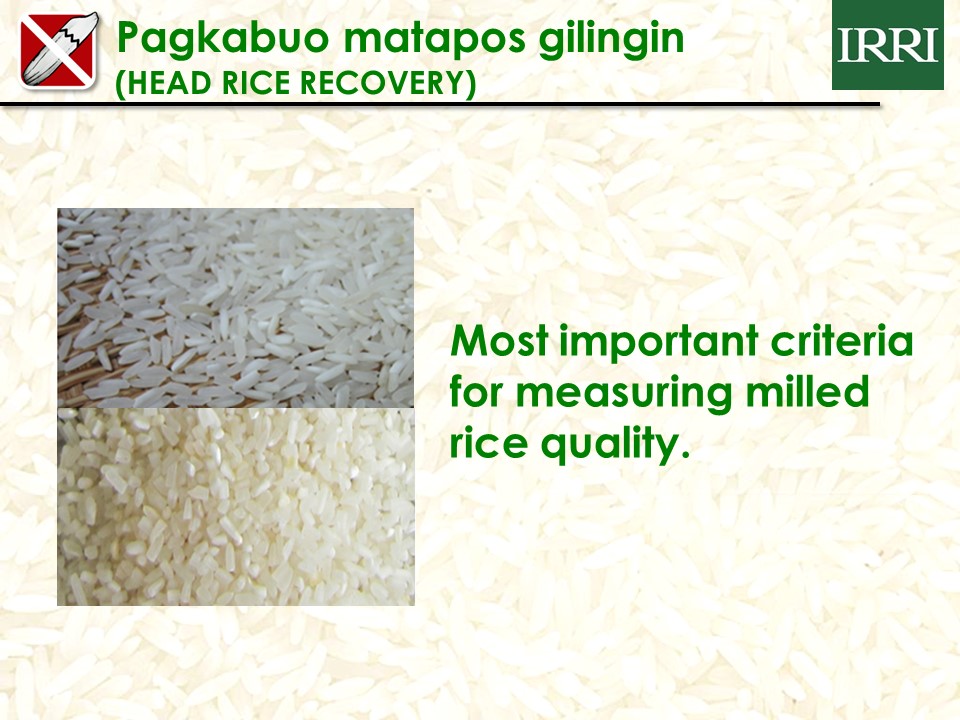(2) |
| --- | --- |
| *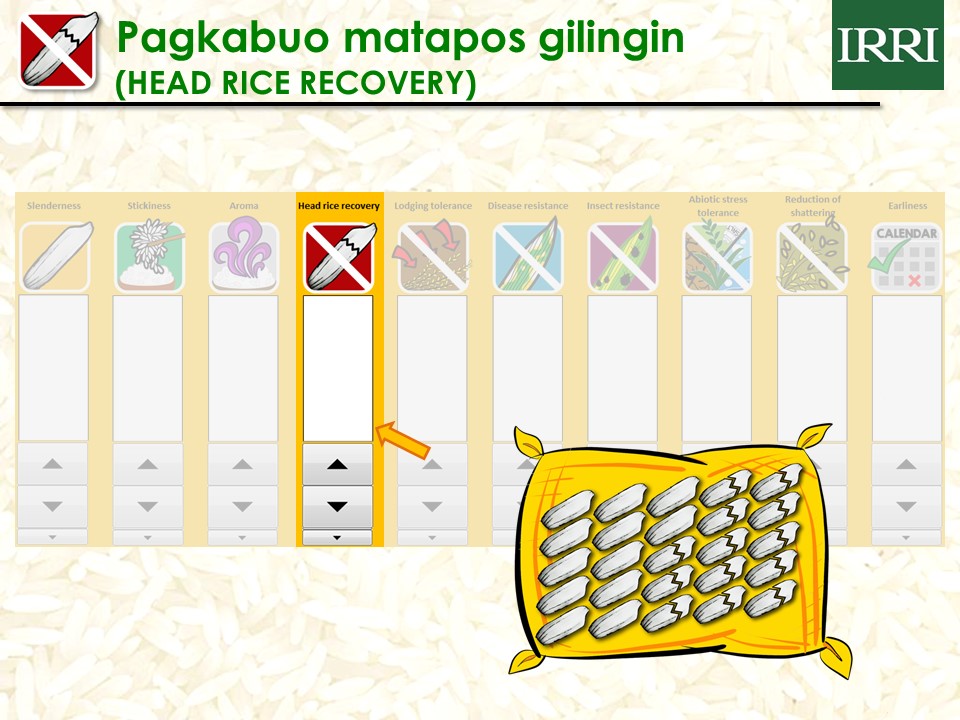*(3) | *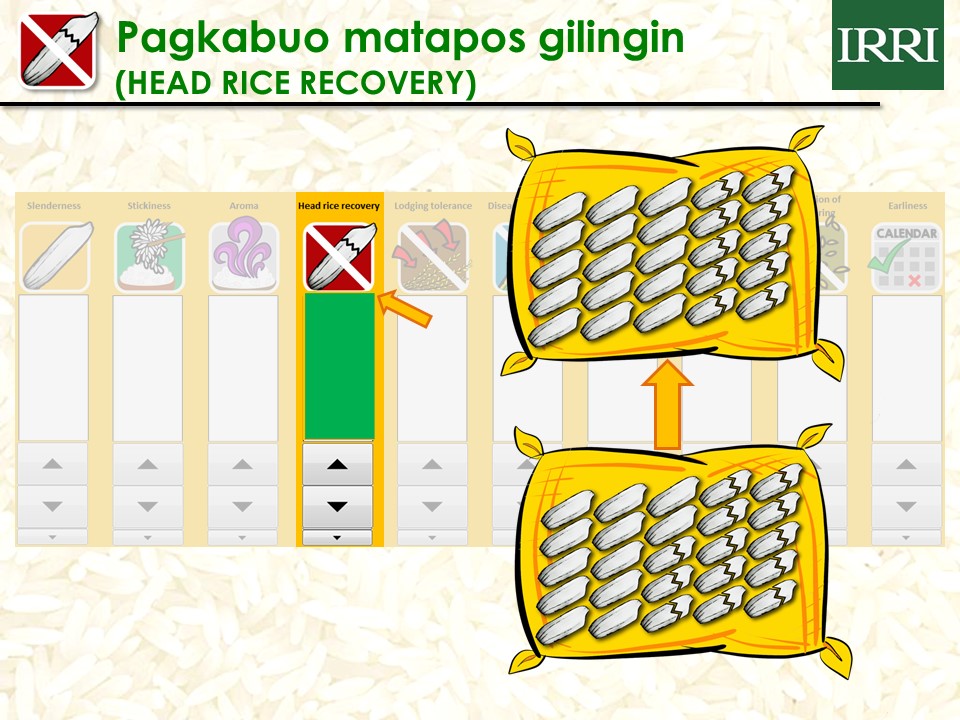*(4) |

Figure 9. Slides used to explain head rice recovery and its VTI levels.

#### **Explain Lodging Tolerance (in local language, in PH: Matibay sa pagdapa):** *This trait of rice variety pertains to the tolerance of rice plants from falling down caused by wind and flood. We all know that it is difficult to harvest lodged rice plants and it reduces your yield. Looking at the image, almost all of the plants were lodged and touching the ground but by increasing the bar up to the middle level, you are increasing the plants that are in upright position. By investing in lodging tolerance at maximum level, you will notice that the majority of the plants are in upright position and few are lodged. If your replacement rice variety, which is mainly for selling, has soft stems and is susceptible to lodging, choose this trait to improve.*

| 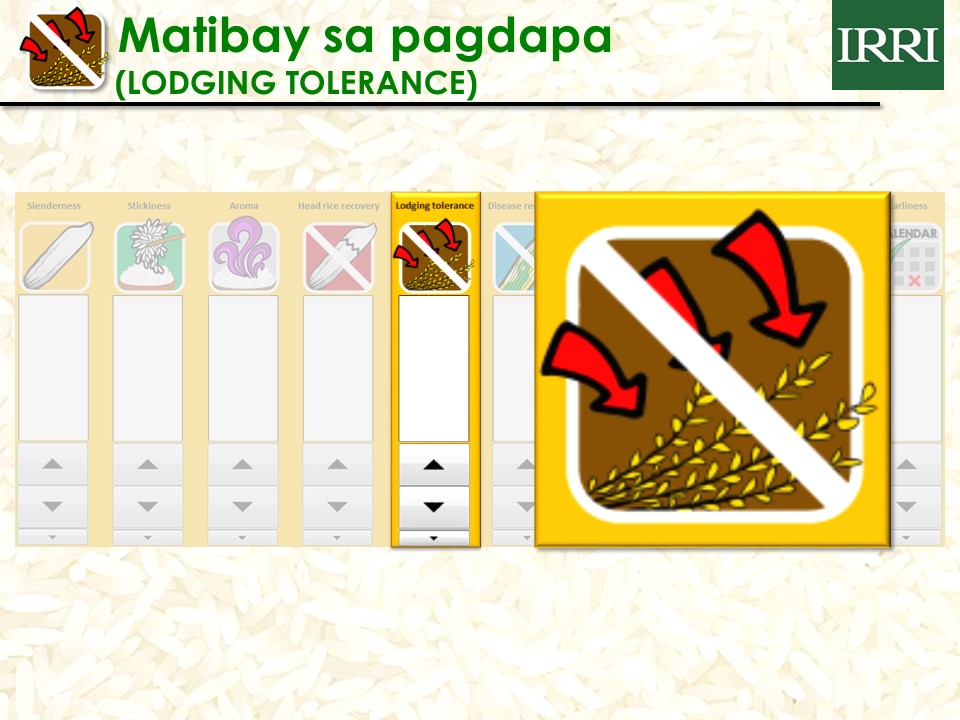(1) | 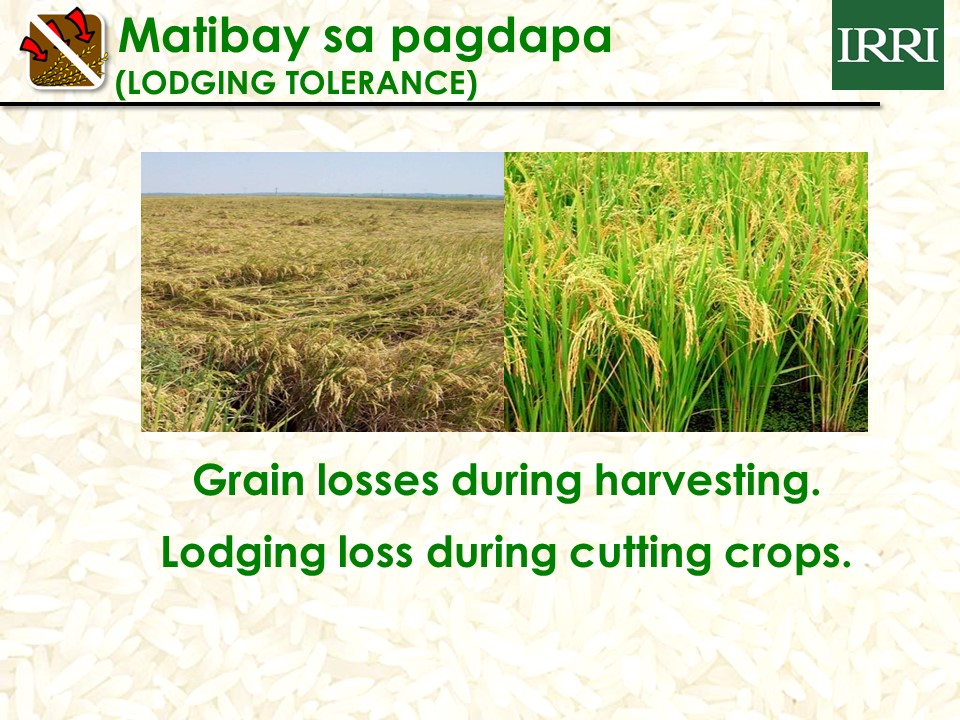(2) |
| --- | --- |
| 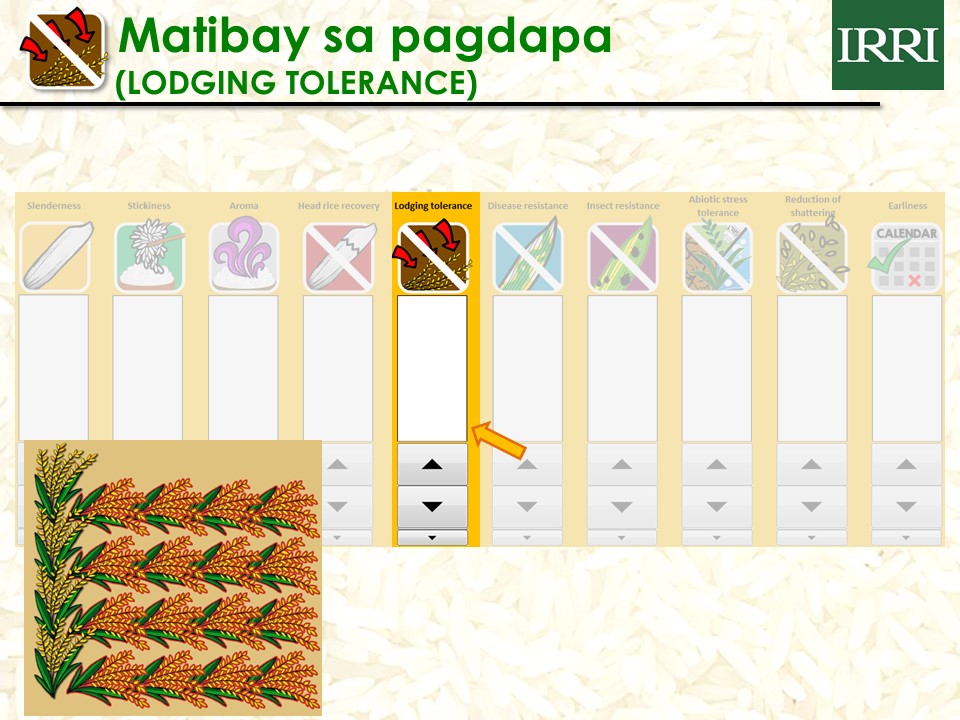(3) | 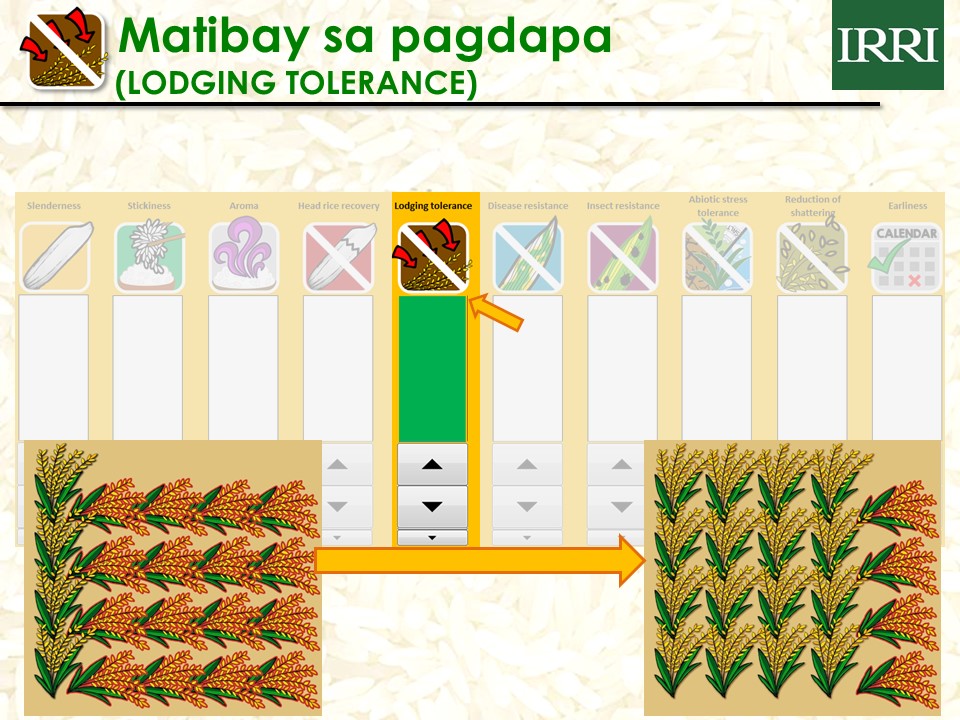(4) |
| 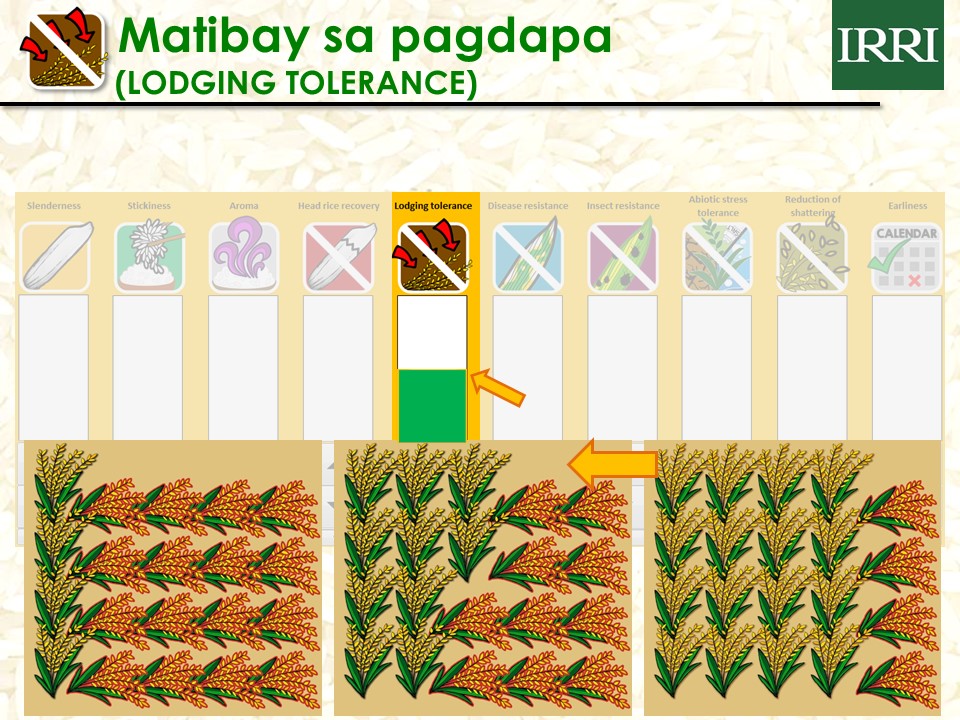(5) | 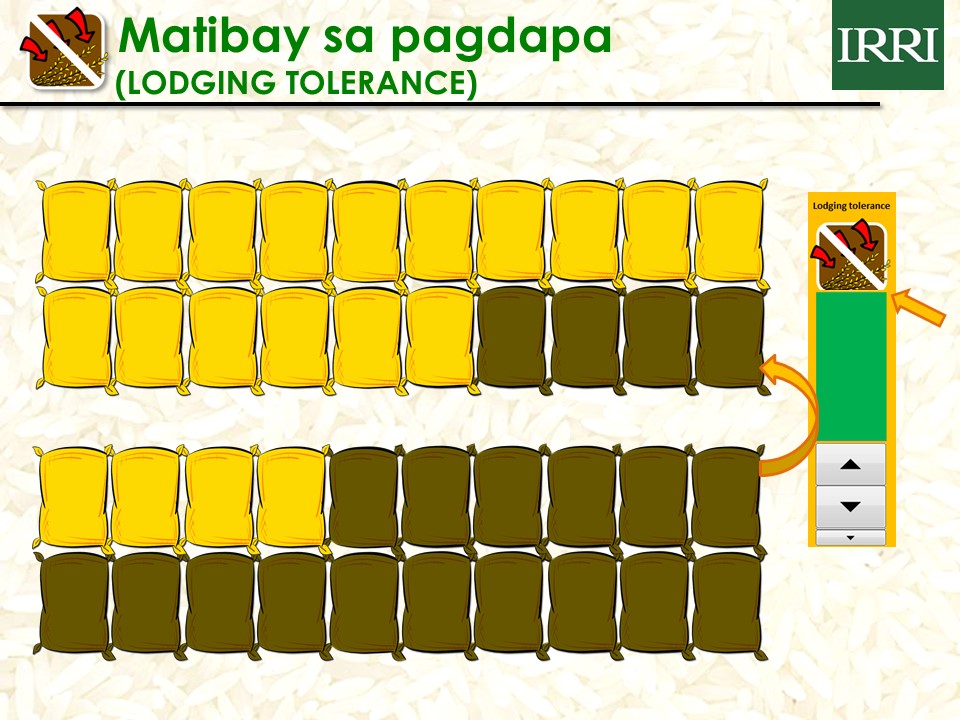(6) |

Figure 10. Slides used to explain lodging tolerance and its VTI levels.

#### **Explain “Disease Resistance” (in local language, in PH: Matibay sa sakit):** *One of the causes of poor harvest in rice is the presence of rice diseases caused by bacteria, viruses and fungi such as* [identify diseases that are more prevalent in the locality] *tungro, blast and bacterial blight. Planting a resistant variety is the simplest and, often, the most cost-effective management for diseases. In the image presented, from a field, which half of it is infected by a disease, by increasing and investing up to its maximum level, the plants will be almost resistant to a disease. If you want to transform your replacement rice variety, which is mainly for selling, into a disease-resistant variety, then increase the bar of this trait.*

| 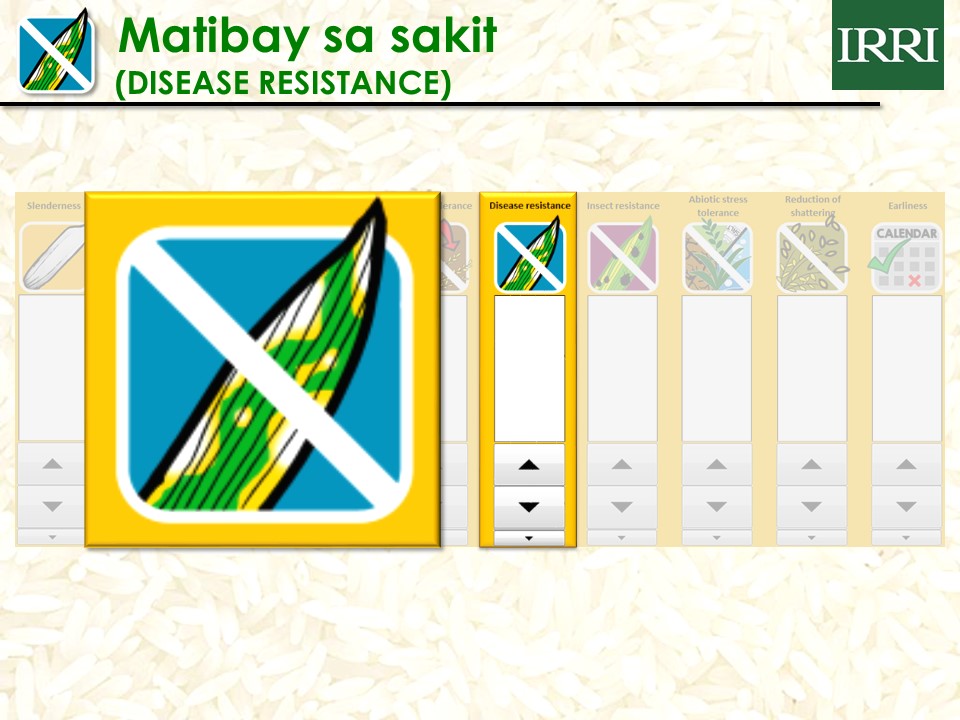(1) | 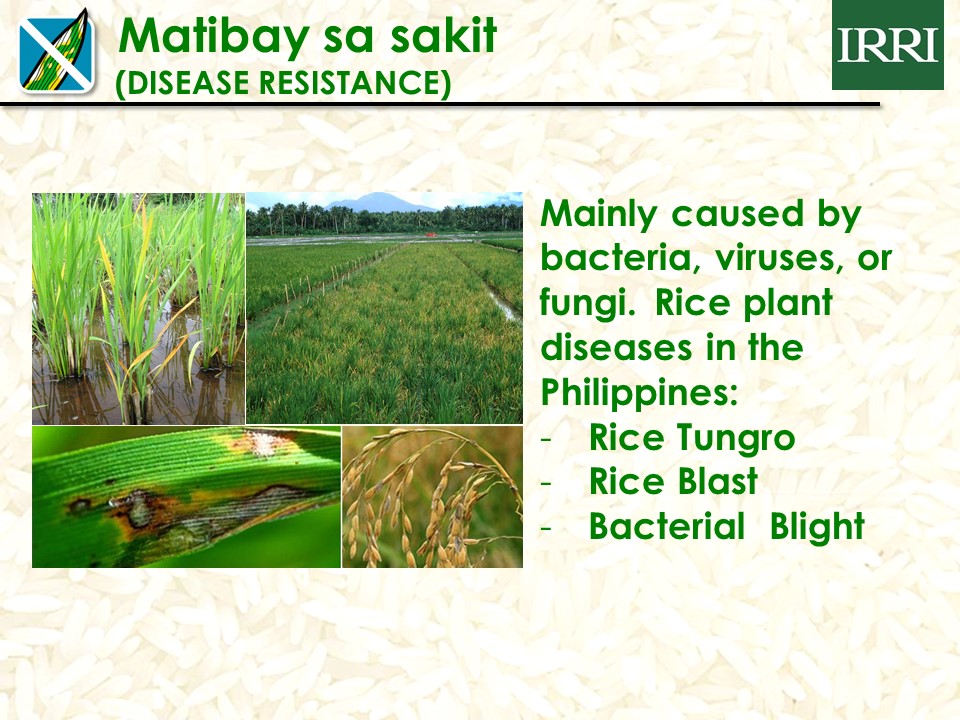(2) |
| --- | --- |
| 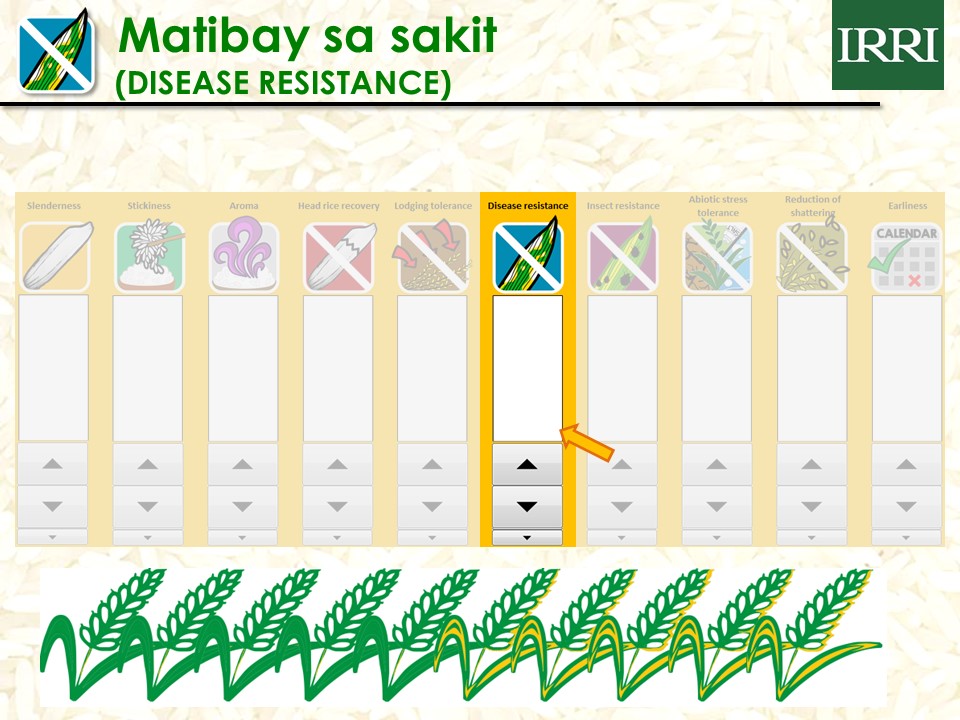(3) | 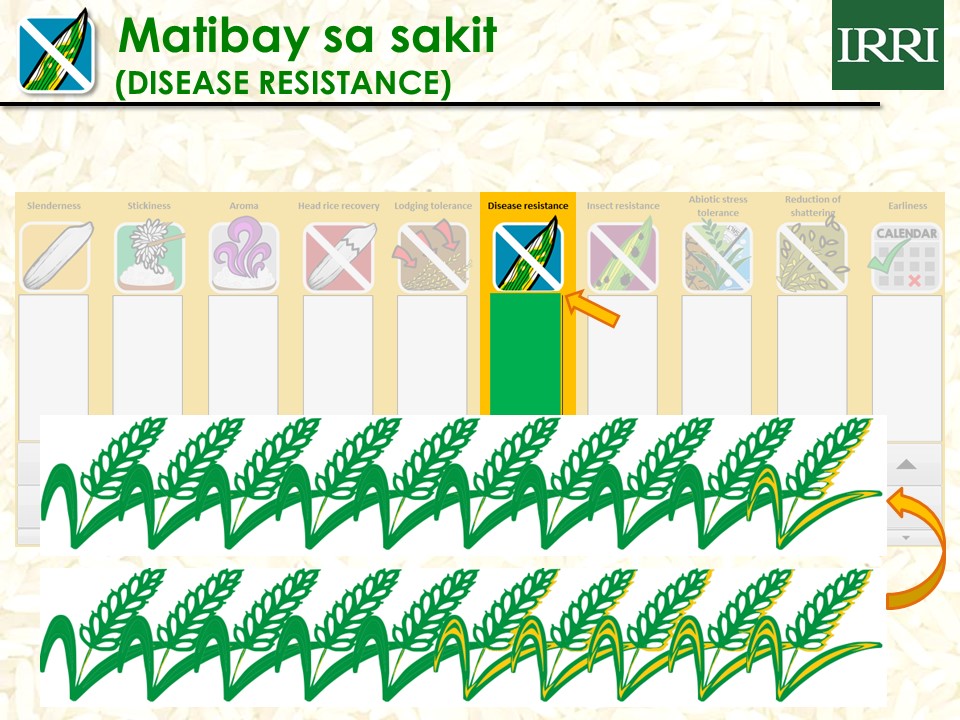 (4) |
| 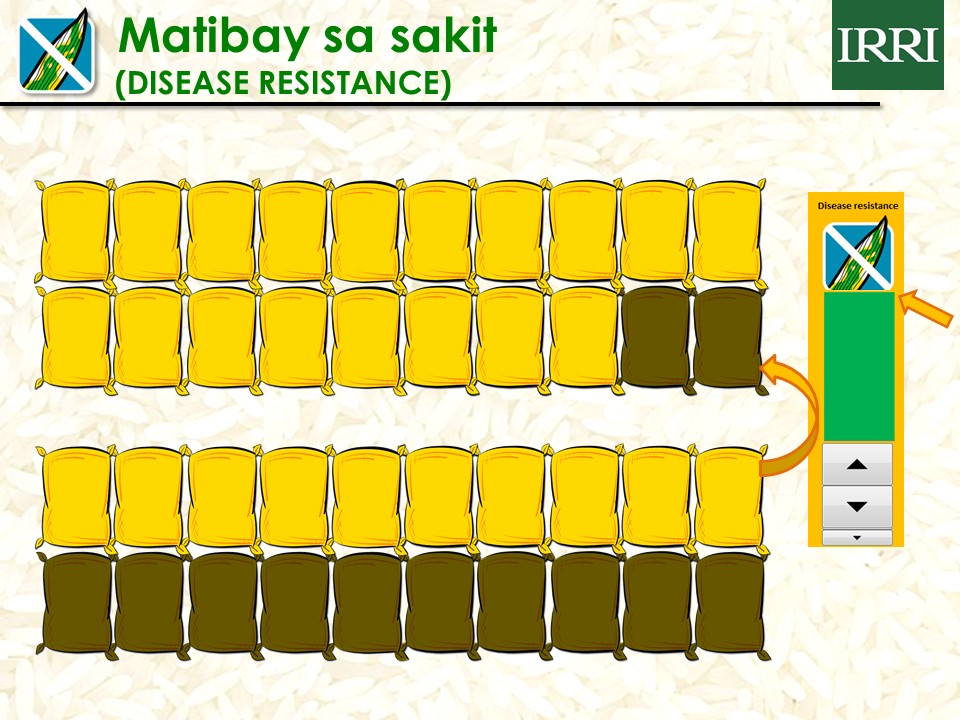(5) |  |

Figure 11. Slides used to explain disease resistance and its VTI levels.

#### **Explain “Insect Resistance” (in local language, in PH: Matibay sa insekto):** *Black bug, stem borer and green leaf-hopper* [name insects that are prevalent in the locality] *are the common insects that inflict significant damage to rice plants in the Philippines which lead to yield reduction. However, we should also remember that not all insects are harmful to rice. There are also some insects that are rice-friendly. The most effective and cost-efficient way of avoiding insect damage is to plant insect-resistant rice varieties. Suppose your replacement variety, which is mainly for selling, is vulnerable to insects like in this image with bugs almost all over the plant and affecting its health, by increasing the bar up to its maximum level, damage due to insects is decreased making it more insect-resistant.*

| 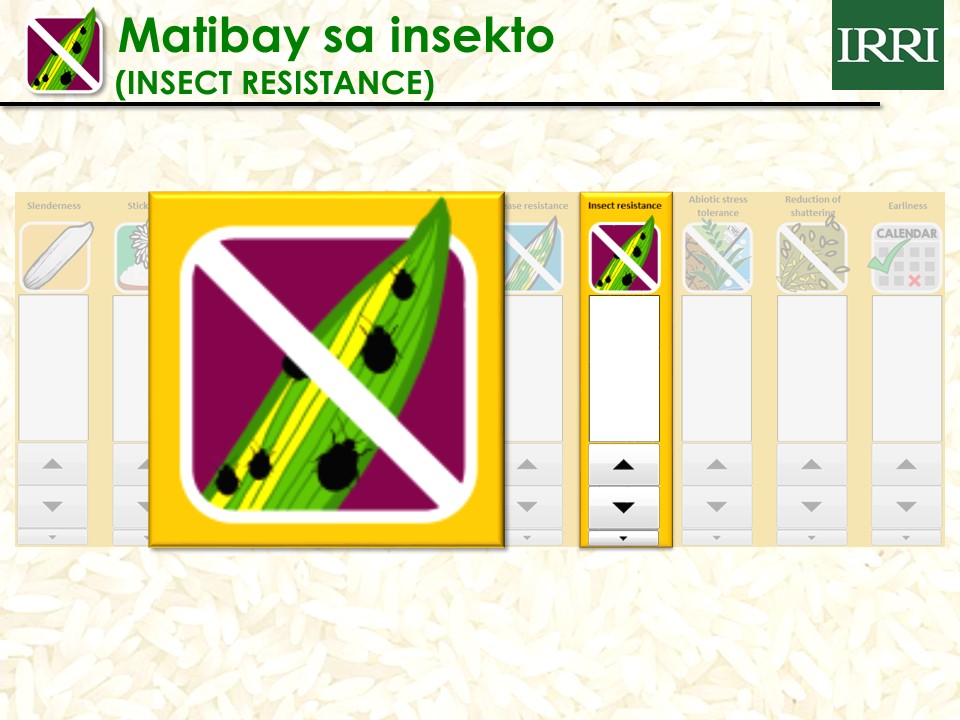(1) | 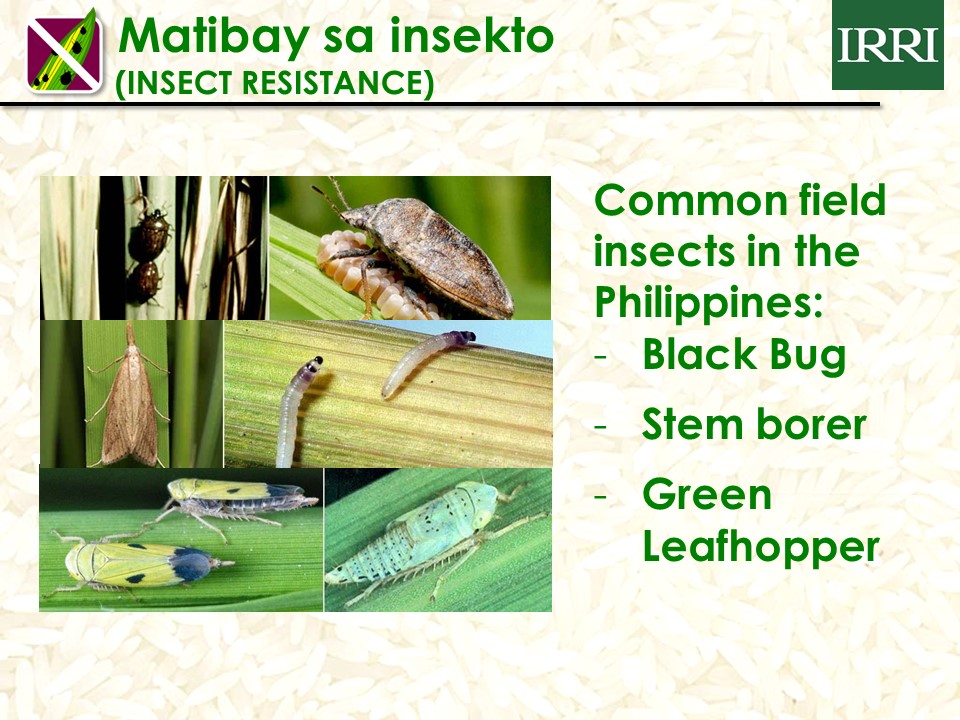(2) |
| --- | --- |
| 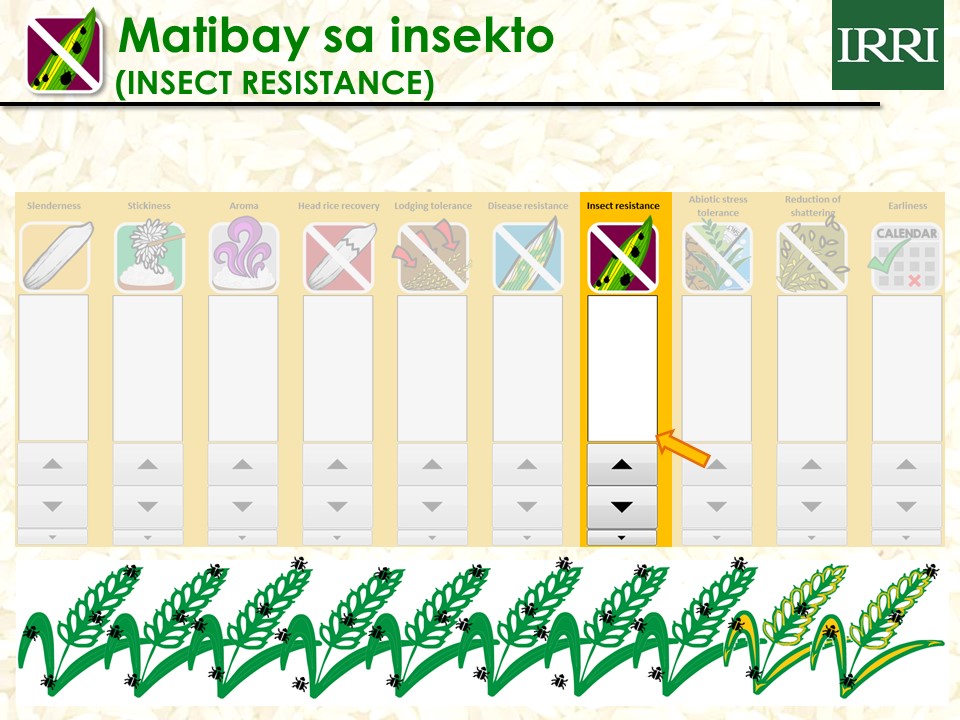(3) | 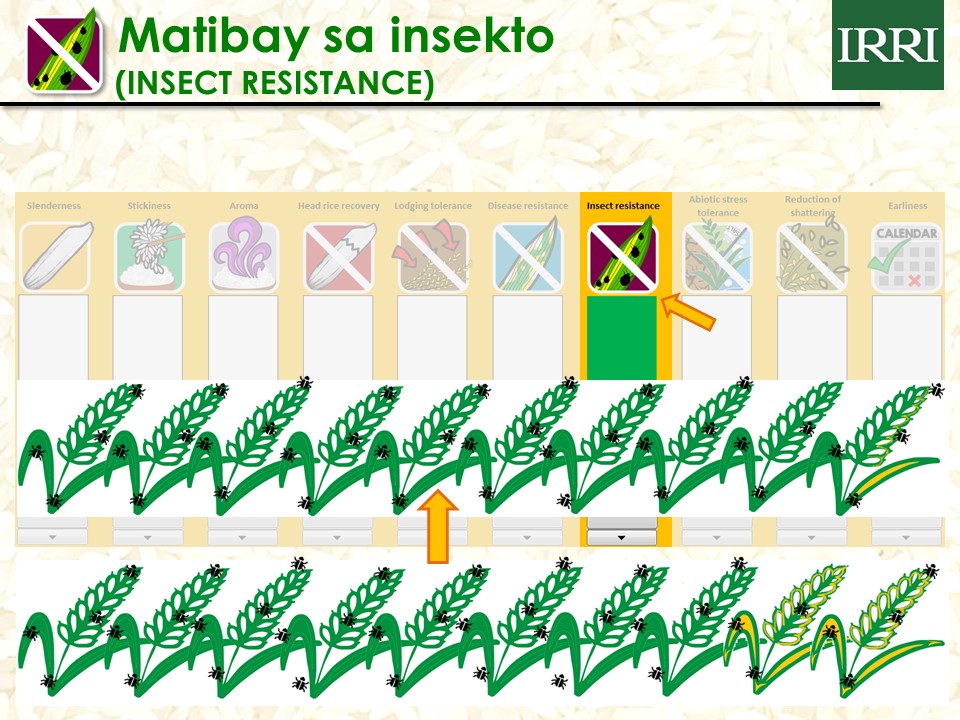 (4) |
| 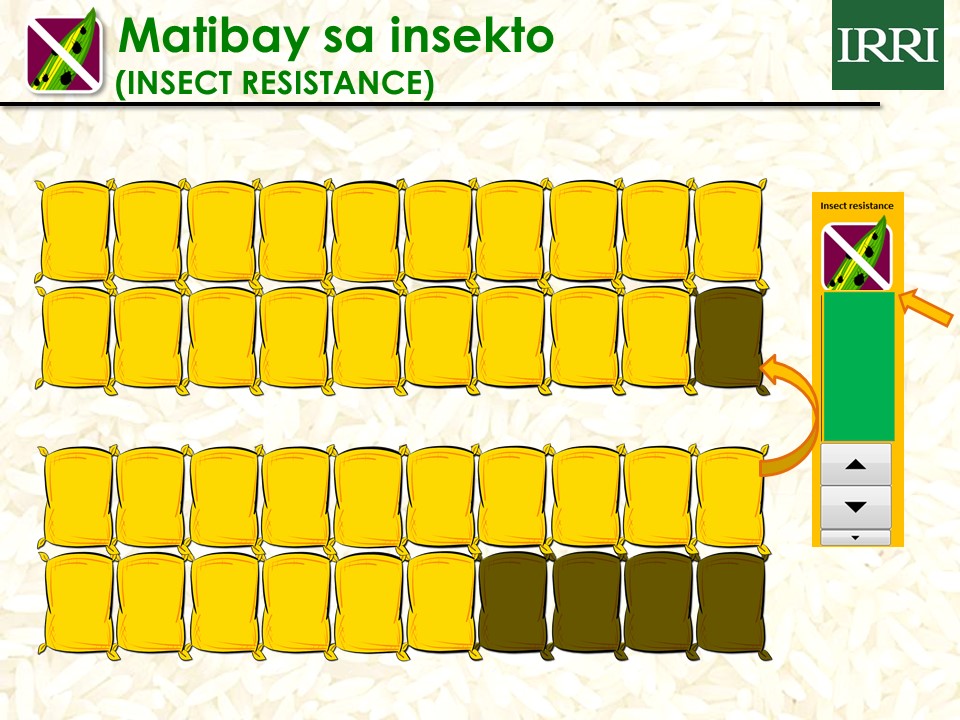(5) |  |

Figure 12. Slides used to explain insect resistance and its VTI levels.

#### **Explain “Abiotic Stress Tolerance” (in local language, in PH: Matibay sa tagtuyot, paglubog, at maalat na lupa):** *This trait is about the tolerance of rice varieties to abiotic stresses such as submergence, drought and salinity. If your paddy field is prone to one of these conditions and your replacement variety, which is mainly for selling, is susceptible to this condition, your variety could be improved to make it more tolerant to abiotic stress by increasing the bar and reaching the maximum level.*

| 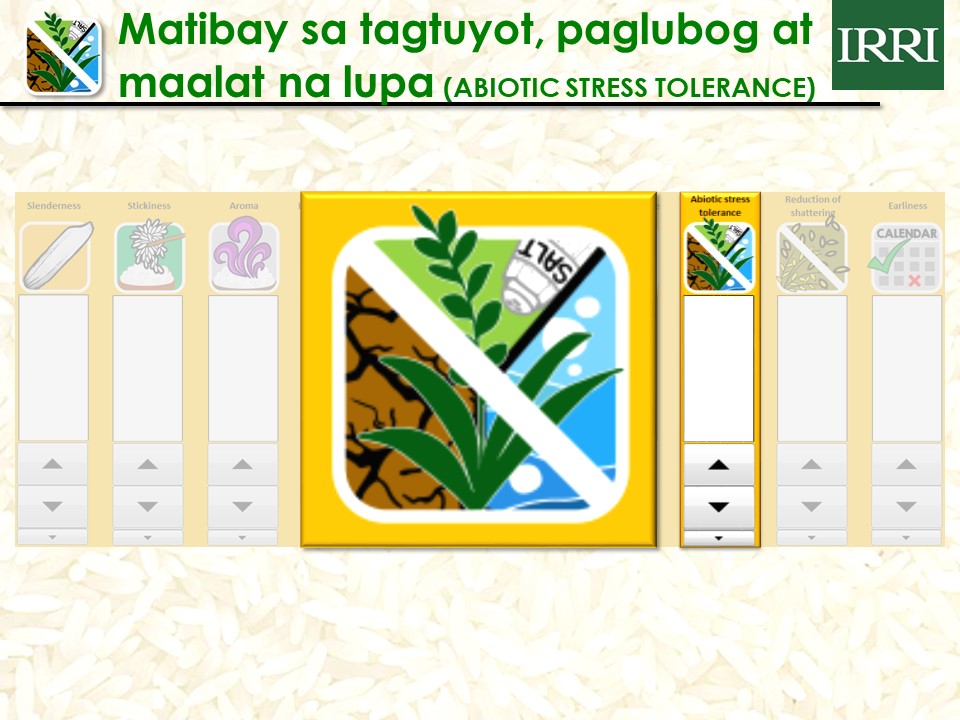(1) | 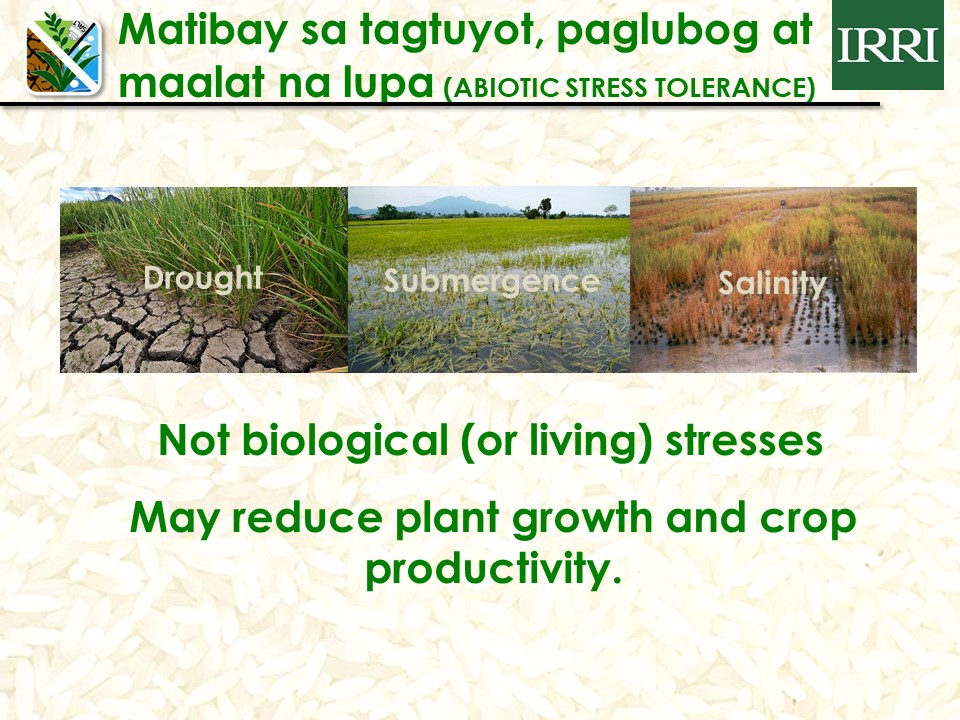(2) |
| --- | --- |
| 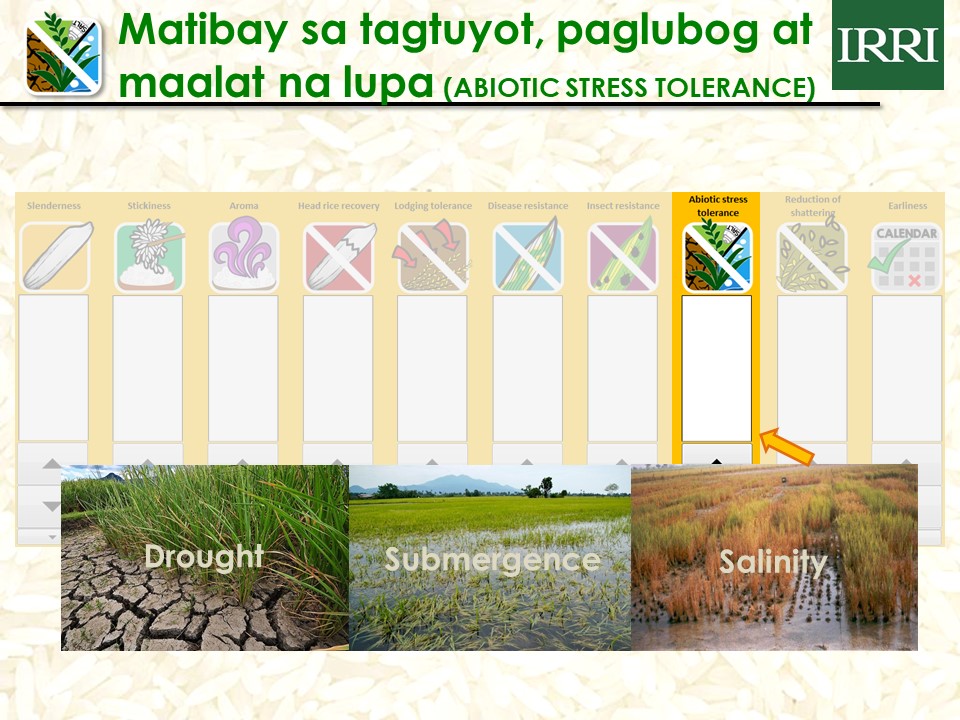 (3) | 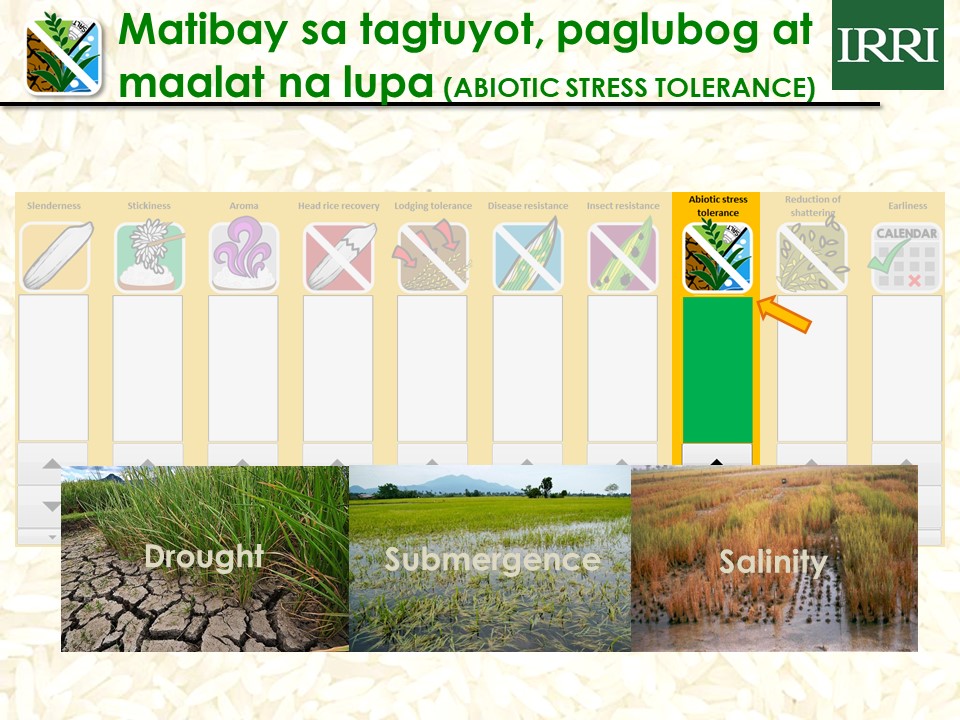(4) |
| 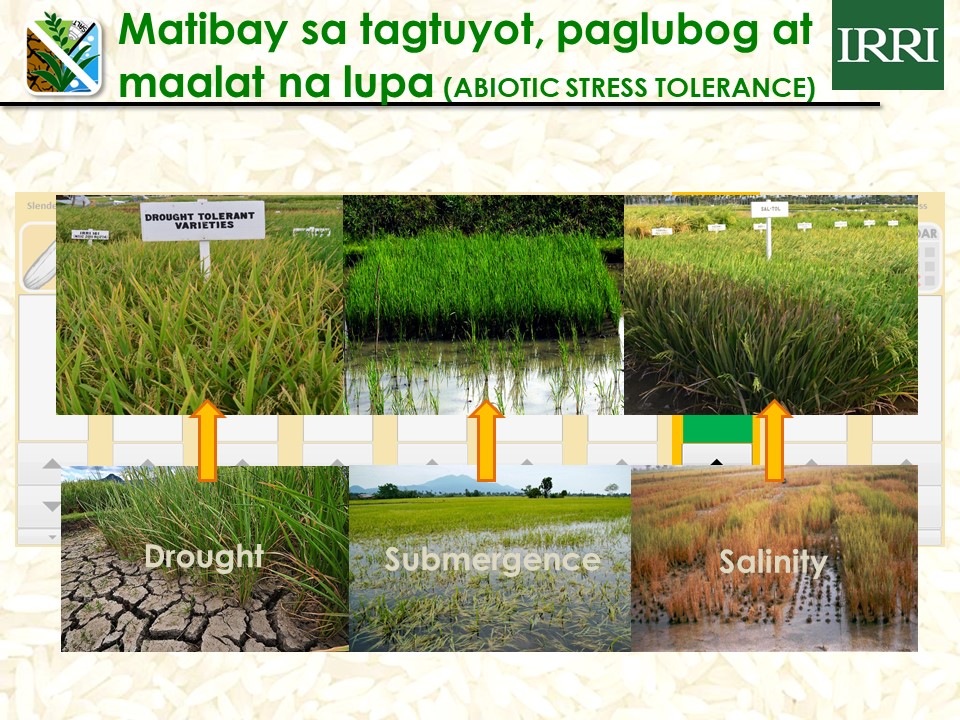(5) | 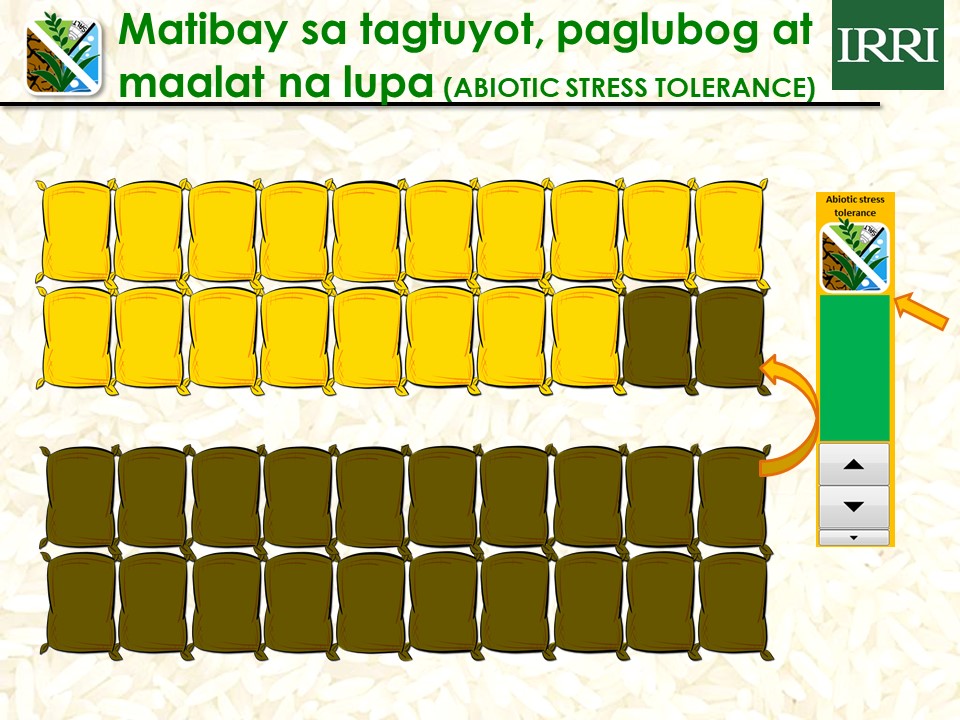(6) |

Figure 13. Slides used to explain abiotic stress tolerance and its VTI levels.

#### **Explain “Reduction of Shattering” (in local language, in PH: Bawas sa panlalagas):** *This rice trait refers to the shedding of mature grains from the panicle caused by birds, wind, rats, and handling. Shattering of grains, somehow, significantly reduces the harvest of our farmers. If the mature grains of your replacement variety, which are mainly for selling, are prone to shattering then select this trait and improve it by increasing the corresponding bar.*

| 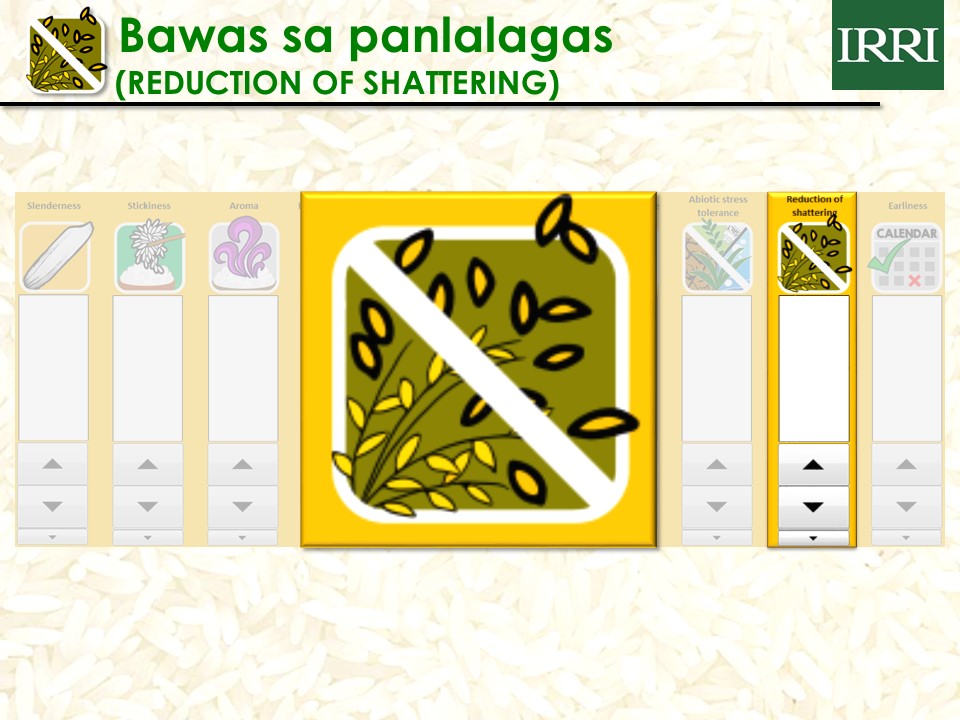(1) | 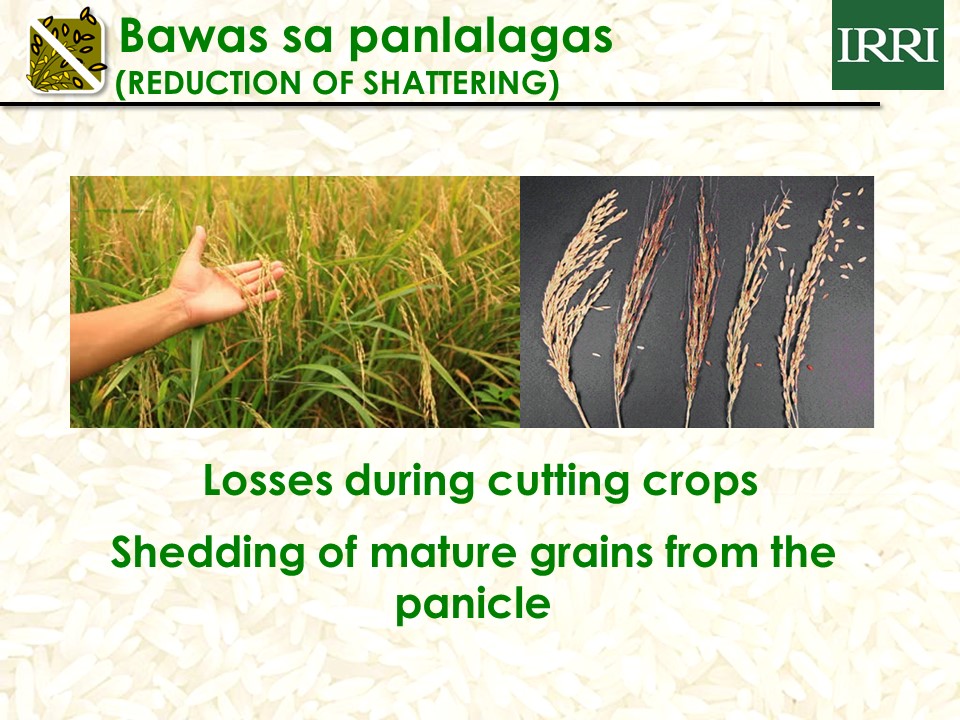(2) |
| --- | --- |
| 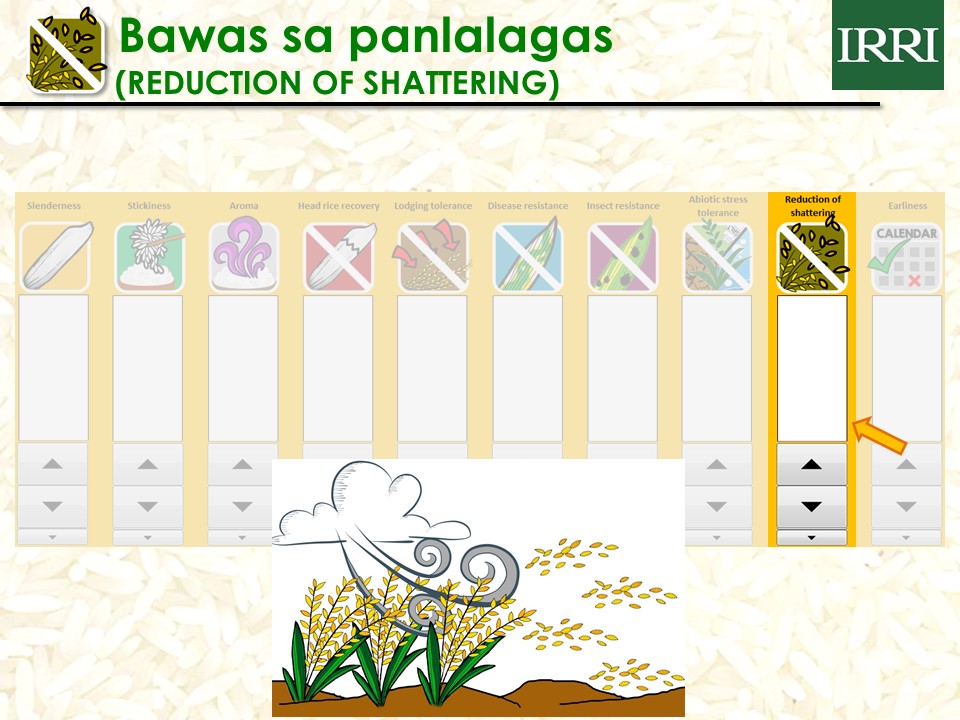(3) | 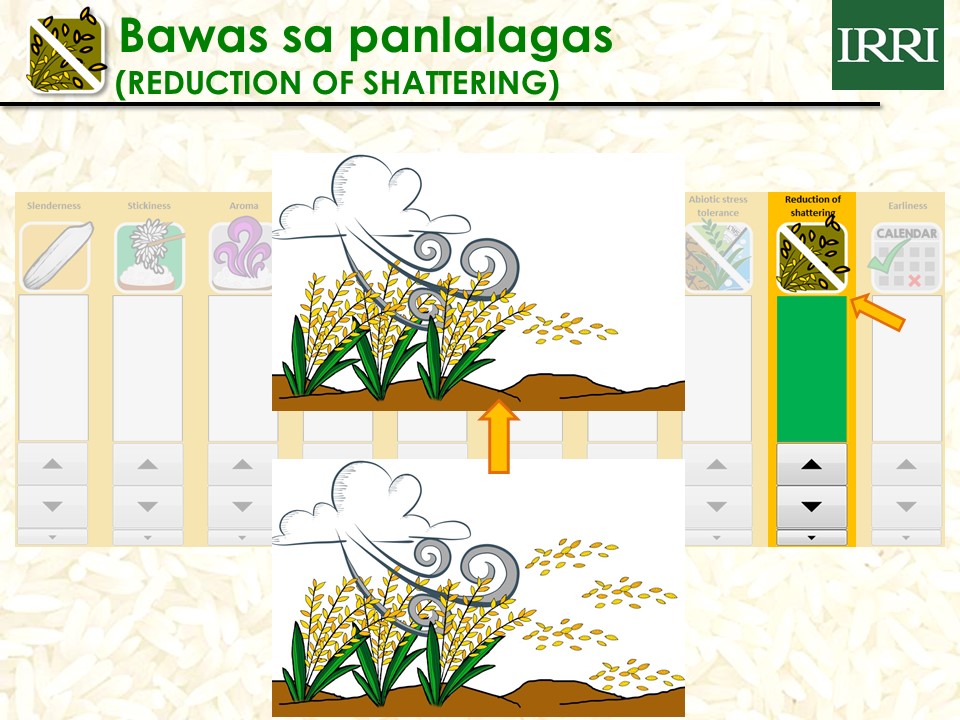(4) |
| 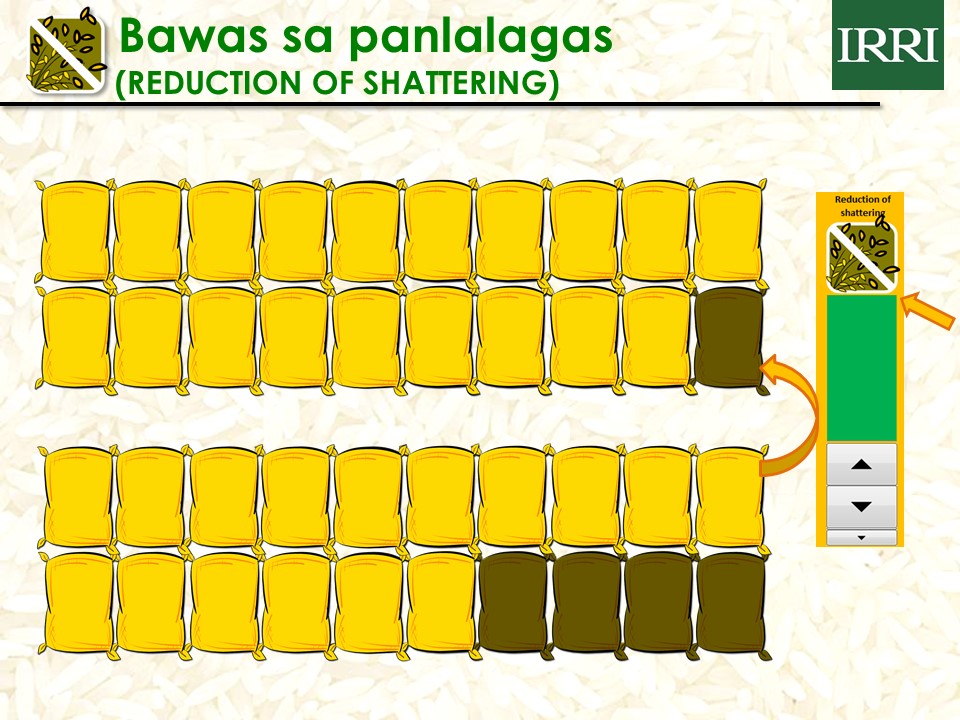(5) |  |

Figure 14. Slides used to explain reduction of shattering and its VTI levels.

#### **Explain “Earliness” (in local language, in PH: Mabilis anihin):** *This trait refers to early maturity of rice. Some farmers want rice varieties that are early maturing so that they can right away start another cropping season or cultivate non-rice crops or to avoid potential monsoon. Suppose your harvest is scheduled at this date* [referring to the calendar in the slide]*, by increasing the bar up to its maximum level, your harvest date will be earlier by 14 days. If there is a need or you want your replacement rice variety, which is mainly for selling, to have shorter maturity duration, then you can choose this trait and increase the corresponding bar up to the number of days you want to save*.

| 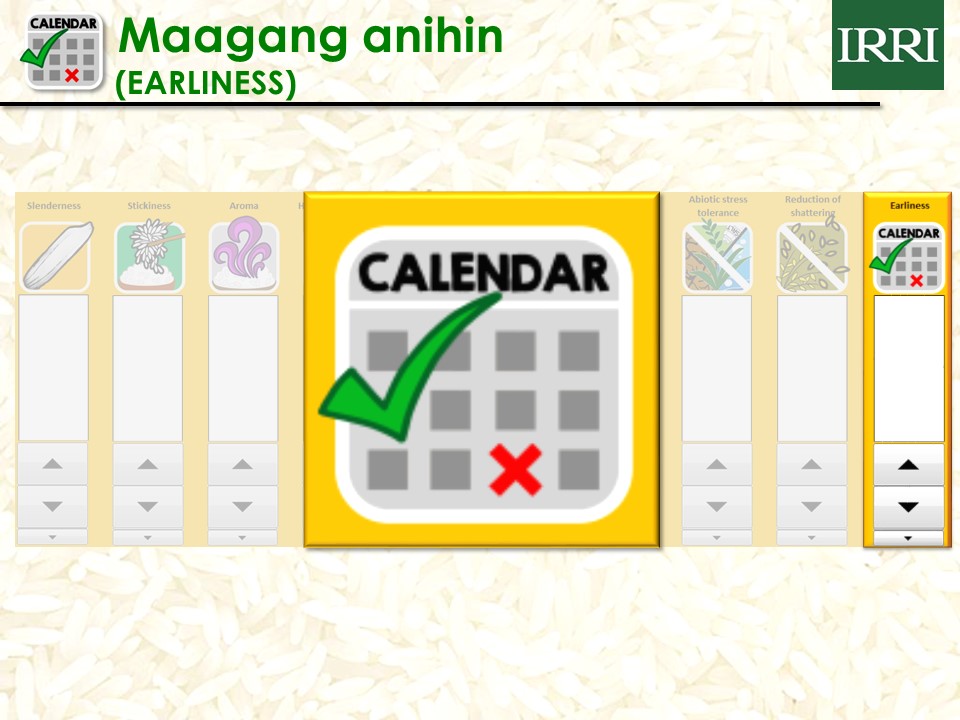(1) | 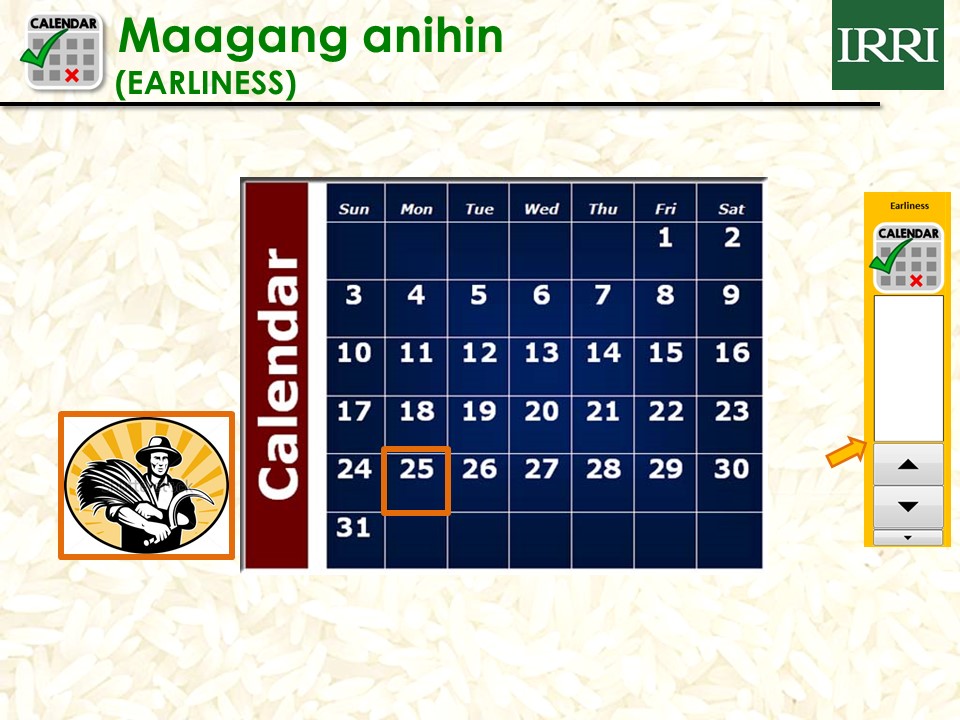(2) |
| --- | --- |
| 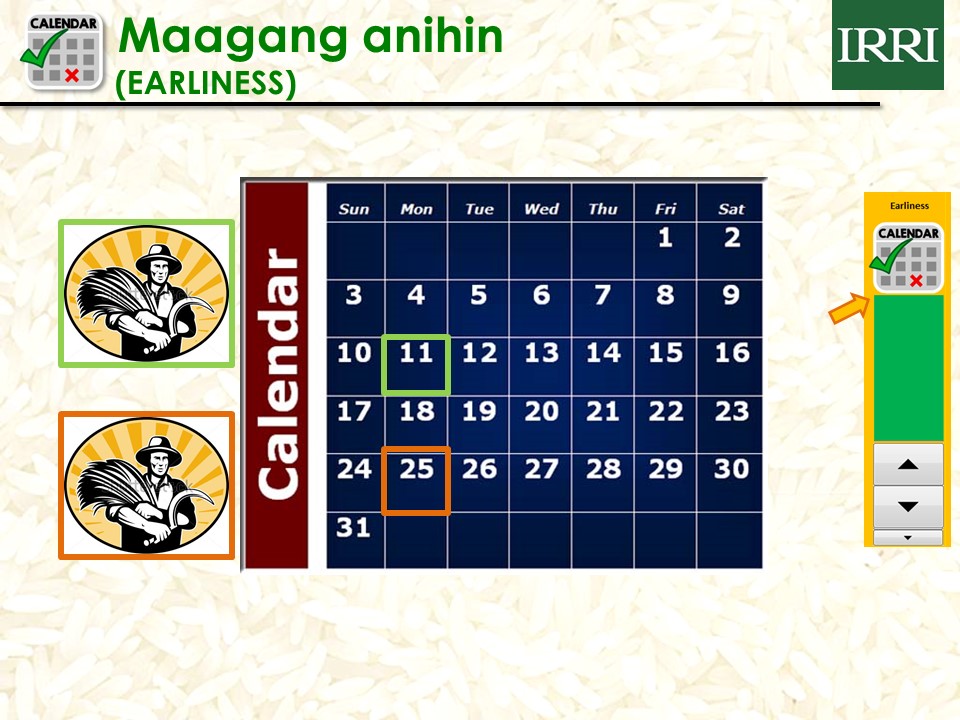(3) |  |

Figure 15. Slides used to explain earliness and its VTI levels.

### Demonstration of the IGA

*And now that you already know the 10 varietal traits and their maximum level of improvements, let us proceed to our task, the investment games. The big donors decided to distribute their grants to farmers. These grants were originally allocated to IRRI’s rice breeding program. Each of you will have an initial amount of 100 PHP, which you can invest in the breeding program that will improve your replacement rice variety to become your ideal variety. As mentioned earlier, the replacement variety can be your most preferred or a popular variety. You may or may have not grown it in the past or currently growing. What is important is that you are familiar with its characteristics and it is the variety that you would like to improve for selling. Before we start the task (investment games), we will ask you to think of the replacement rice variety that you would like to improve for selling. Then, choose one or multiple traits among the 10 varietal traits that you want to add or to improve in this particular variety given the limited budget of 100 PHP.*

#### **Single trait – demonstrate that as you improve a trait, the budget decreases and the risk increases accordingly:** *As you increase the level of improvement of a trait, a corresponding cost will be deducted from your budget and a relative investment risk will be added. The initial improvement of a trait is more expensive compared to the succeeding levels because of the fixed cost. The first level of new trait improvement is costly since opening a new research program for this trait will require initial expenses such as establishing a new laboratory and field experiments.*

Improvement of one trait will not affect other traits (*ceteris paribus*): *Improving a trait will not affect the level of other traits.*

#### **Trait combination – demonstrate that once you improve an additional trait, the initial improvement is costly compared to the succeeding improvement because of the fixed cost**: *Adding a new trait will initially incur a greater amount of costs compared to the succeeding levels and this is due to fixed costs.*

Traits are synergistic: *There are combinations of traits that are cheaper to develop in one breeding program compared to separate single-trait breeding programs.*

A particular trait combination is antagonistic or you may choose not to explain this to avoid causing bias against these two traits: *There are some traits that are counteracting each other. A good example is slenderness and head rice recovery. The more slender the rice, the lesser its head rice recovery since slender rice is brittle and prone to breaking during milling. This is the reason why the improvement of these traits in a single variety is more costly than producing two separate varieties, i.e. one with high slenderness and the other with high head rice recovery.*

#### **Explain “status quo” (opt out) and possible returns**

We explained to participants that they were not obliged to invest their share and that they could keep any amount they desired and take it home for other uses. However, we also told them that breeding generated, on average, a 10-fold return to investment (following Fan et al., 2005). Hence, the perceived penalty for not investing would be an opportunity cost of the certainty equivalent of around 10 times the endowment they received or 1,000 PHP. Given the riskiness of the investment, farmers could lose as much as 100 PHP (in case farmers spent their entire endowment and all selected VTIs failed and generated zero returns leading to farmers having to reimburse their fund) and gain as much as 2,300 PHP.

We also mentioned that the duration of breeding a new rice variety was around six years. However, we explained that for the task at hand, we would fast-forward the breeding process so that after they invested, we could instantaneously compute and pay out the return. We explained that the return on investment was based on experts’ computation and simulation.

## The IGA Experiment

Before starting the experiment, participants were informed of the number of rounds they would go through in the investment games. They were told that they would first play the IGA independently and simultaneously for two target seasons, the wet and the dry. They would then play the IGA jointly for two target seasons as well. In each season, the participants worked on the traits of their replacement variety they wanted to improve.

At the start of the experiment, experimental ‘agents,’ working with the facilitators as part of the research team, collected their assigned tablets and accompanying sheets and then approached their designated rows and tables. Agents’ tasks were to assist in introducing the IGA to farmer-participants and to make things comfortable for them.

To avoid intrahousehold influence, agents for husbands and wives moved simultaneously from one row to another. To capture the priority of each gender and provide equal opportunity for the husband and the wife, agents for the joint session had two stylus pens and were instructed to place the tablet in the middle of the table. They were also asked to observe the attitude of the husband and wife and record who dominated discussions.

While some participants were completing the experiment, a recruiter and enumerators, respectively, implemented participant registration and worked with participants to complete a survey questionnaire. This strategy helped to reduce the time of the whole experimental process and minimized the idle time of the participants who were not yet administered the IGA by the experimental agents.

The survey questionnaire collected information on the farming household, including constraints and the time and risk preference of the respondents. Participants were also asked to indicate if they understood the investment game. Specifically, in the post-experiment survey, two review questions were asked to test participants’ understanding of the IGA (Figure 16). In the first question, participants’ understanding of the riskiness was tested. Two VTIs were shown with different degree of riskiness and participants were asked for which VTI the probability of success is highest. In the second question, participants’ understanding of the value of prioritization was tested.


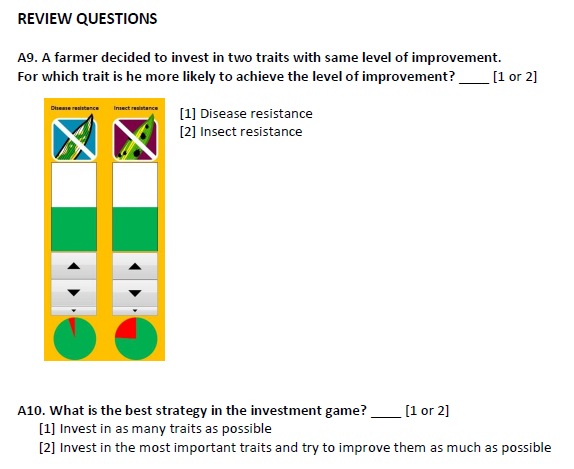


Figure 16. The two questions asked to test whether participants understood the investment game.

## Closing ceremony

After all participants finished all six rounds of the IGA, one participant was requested to roll a dice to randomly determine the binding round among the six rounds (Round A – husband /wet season, Round B – husband/dry season, Round C – wife/wet season, Round D – wife/dry season, Round E – joint/wet season, Round F – joint/dry season). The binding round was the basis for paying out the random returns; and, as a result, each household received only one payment depending on which round was determined by the dice to be binding. Each household’s file for the binding round was opened and the random return was paid in cash to the participants.

Before we closed the session, we thanked all participants and assured them that all of the information they shared with us would be relayed to the IRRI rice breeders. Finally, we asked the participants to take a group photograph.

**References**

Custodio, M.C., Demont, M., Laborte, A., Ynion, J., 2016. Improving food security in Asia through consumer-focused rice breeding. Global Food Security 9, 19–28.

Demont, M. and Villanueva, D., 2019. Eliciting multitrait cost functions in plant breeding through competitive tenders. International Rice Research Institute (IRRI), Los Baños, Philippines.

Fan, S., Chan-Kang, C., Qian, K. and Krishanaiah, K., 2005. National and international agricultural research and rural poverty: The case of rice research in India and China. Agricultural Economics 33, 369–379.

1. Farmers were provided information on the top most important rice traits considered by consumers in Metro Manila, which is a major market for the rice produced in Nueva Ecija. The top five rice traits are: (i) aroma; (ii) white colour grains; (iii) soft texture; (iv) volume expansion; and (v) chewy texture. They were also provided information on the most preferred rice “variety” in Metro Manila, which are: (i) Sinandomeng (23% market share); (ii) Jasmine rice (21%); (iii) Dinorado (19%); (iv) Angelika (19%); and (v) others (18%). [↑](#footnote-ref-1)
2. It was explained to the participants that temperatures are likely to rise, which can cause severe drought, ice caps melting, and extreme precipitation. It was also mentioned that the frequency of extreme weather events is likely going to rise, which can lead to more frequent droughts or floods, and can cause more uncertainty on when the rainy season starts. [↑](#footnote-ref-2)
